# Social evaluation of skilfulness in Tonkean macaques (*Macaca tonkeana*) and brown capuchins (*Sapajus apella*)

**DOI:** 10.1007/s10071-025-02039-9

**Published:** 2026-01-14

**Authors:** Marie Hirel, Michele Marziliano, Hélène Meunier, Hannes Rakoczy, Julia Fischer, Stefanie Keupp

**Affiliations:** 1 Department of Primate Cognition, Georg-August-Universität Göttingen, Johann-Friedrich-Blumenbach Institute, Kellnerweg 4, 37077 Göttingen, Germany; 2 Cognitive Ethology Laboratory, German Primate Center, Leibniz Institute for Primate Research, Kellnerweg 4, 37077 Göttingen, Germany; 3 Department of Molecular Medicine, Sapienza University of Rome, Rome, 00161 Italy; 4 Centre de Primatologie de l’Université de Strasbourg, Chemin du Fort Foch, Niederhausbergen, 67207 France; 5 Laboratoire de Neurosciences Cognitives et Adaptatives, UMR 7364, 12 rue Goethe, Strasbourg, 67000 France; 6 Leibniz ScienceCampus, German Primate Center – Leibniz Institute for Primate Research, Kellnerweg 4, 37077 Göttingen, Germany; 7 Department of Cognitive Developmental Psychology, Georg-August-Universität Göttingen, Georg-Elias-Müller Institute for Psychology, Waldweg 26, 37073 Göttingen, Germany

**Keywords:** Competence, Skills, Primates, Social information sampling, Social decisions, Looking time

## Abstract

For optimal decision-making, social animals can benefit from evaluating others’ behaviours. Some species seemingly consider the skills of others when deciding who to interact with in different contexts. Yet, whether and how nonhuman animals form impressions about others’ competence is still unclear. In this study, we investigated whether Tonkean macaques (*Macaca tonkeana*) and brown capuchins (*Sapajus apella*) can evaluate the skilfulness of others. Subjects observed two human actors (one skilful, one unskilled) trying to open several food containers. Only the skilful actor successfully opened the containers and released food so the experimenter could give it to the subjects. Our results revealed that subjects did not choose the skilful actor significantly more frequently than the unskilled one. Their choices for the skilful actor did not increase through trials nor were they based on the outcomes experienced in previous trials. However, when we considered their initial preferences for the human actors, we observed a significant shift in preference for the skilful actor. Our subjects also looked preferentially at the skilful over the unskilled actor when both simultaneously manipulated a container. While the underlying cognitive mechanisms (impression formation vs. outcome-based process) are still unclear, our findings indicate that Tonkean macaques and brown capuchins may have used social information about the actors’ skills to inform their decisions and raise questions about which behavioural measures best capture social evaluation in nonhuman species.

**Supplementary Information:**

The online version contains supplementary material available at 10.1007/s10071-025-02039-9.

## Introduction

Forming impressions about others from their past behaviours is a valuable cognitive skill, for example, in social contexts that require choosing interaction partners or role models (Corriveau et al. 2009; Rakoczy et al. 2009; Fusaro et al. 2011; Kushnir et al. 2013; Hermes et al. 2016, 2017; Wu et al. 2016). Most research has focused on evaluating others’ prosocial characteristics in nonhuman primates (Russell et al. 2008; Subiaul et al. 2008; Herrmann et al. 2013; Anderson et al. 2013a, b, 2016; Kawai et al. 2014) and a few other animal species (dogs and wolves: Kundey et al. 2011; Jim et al. 2022; fishes: Bshary and Grutter 2006; Vail et al. 2014; elephants: Jim et al. 2021; cats: Leete et al. 2020). Social evaluation of others’ skills (i.e., the performance of an individual in a particular task; Sih et al. 2019) is also essential social information to consider when deciding who to interact with. Individuals might benefit from observing and/or interacting with the skilful foragers of their group to access food resources or to learn new foraging skills (although skilful foragers may also represent a threat or competitor for food access, making interactions risky). Similarly, recruiting the most skilful partners seems advantageous for successful cooperation.

Some field experiments show that several nonhuman primates increased their affiliative behaviours toward the only group members skilled at providing food in a novel experimental foraging situation (Stammbach 1988; Fruteau et al. 2009; O’Hearn et al. 2025b; Karakoç et al. 2025). Others preferentially observed (Ottoni et al. 2005; Tan et al. 2018) or copied (Horner et al. 2010; Kendal et al. 2015; Canteloup et al. 2021) the most skilful group members at a foraging technique. However, only a few experimental studies investigated the ability to evaluate the skills of others in nonhuman species. After observing two human actors repeatedly trying to open containers filled with food, long-tailed macaques (*Macaca fascicularis*) preferentially chose (Placì et al. 2019), and dogs preferentially looked at or approached (Piotti et al. 2017; Chijiiwa et al. 2022) a skilful over an unskilled actor. Chimpanzees (*Pan troglodytes*) and coral trout (*Plectropomus leopardus*) strategically recruited the most skilful conspecific partners for cooperation (Melis et al. 2006; Vail et al. 2014; Keupp and Herrmann 2024).

These previous studies suggest that some animal species can consider the skills of others and behave differently towards individuals who differ in their degree of skilfulness. Yet, the cognitive processes underlying these behaviours are still unclear. Individuals can adapt their behaviour toward skilful foragers because they make inferences about the partner’s traits and attribute competence to them (i.e., *impression formation process*) or because they learn to associate the foragers’ actions with the resulting outcome (i.e., *outcome-based process*; for a review on social evaluation and underlying cognitive mechanisms, see O’Hearn et al. 2025b). For example, when recruiting conspecifics for cooperative tasks, chimpanzees appeared to engage in a Win-stay/Lose-shift strategy, making decisions based on the outcome of the previous test trial rather than on an evaluation of their partners’ skills (Melis et al. 2006). In addition, confounding factors such as individual differences other than skills, familiarity, or pre-existing relationships between the subjects and the individuals being assessed were not always considered in previous studies. To broaden our understanding of social evaluation in nonhuman primates, we investigated whether two monkey species, Tonkean macaques (*Macaca tonkeana*) and brown capuchins (*Sapajus apella*), can evaluate the skilfulness of others at making food accessible.

Both species live in multi-male multi-female societies characterised as tolerant (Thierry et al. 1994, 2004; De Waal 2000; Fragaszy et al. 2004; Thierry 2007; Riley 2010). They demonstrated social cognitive skills such as social relationships knowledge, attention and goal-directed understanding, and visual perspective-taking (Fragaszy et al. 2004; Phillips et al. 2009; Defolie et al. 2015; Canteloup et al. 2016, 2017; Canteloup and Meunier 2017; Whitehouse and Meunier 2020). They exhibit tool-using in foraging contexts (Anderson 1985; Fragaszy et al. 2004; Ducoing and Thierry 2005; Ottoni and Izar 2008), and brown capuchins even showed preferential attention toward skilful conspecific foragers (Ottoni et al. 2005). Tonkean macaques had never been tested on social evaluation before (except in Hirel et al. 2025a) while brown capuchins already demonstrated social evaluation of human actors’ helpfulness and reciprocity (Anderson et al. 2013a, b). Therefore, testing brown capuchins, a species for which we already have observational and experimental evidence of potential social evaluation abilities, allows us to better gauge the strengths and limits of our experimental paradigm by assessing both species’ results (e.g., methodological explanations or species-specific differences). While we hypothesised that both species could evaluate others’ skills and did not aim to compare species directly, any observed differences can provide valuable theoretical and methodological insights for future research.

In this experiment, subjects could sample information about two human actors’ skills from observation during two demonstration sessions. One actor (skilful) always succeeded at opening transparent containers filled with food, while the other actor (unskilled) always failed. Subjects received a food reward from a third person (the experimenter) once a container was successfully opened by the skilful actors, whereas they went empty-handed after each unsuccessful attempt by the unskilled actor. Then, the subjects completed a Choice Test and two Expectation Tests. In the Choice Test, we measured the proportion of choosing the skilful actor. We predicted that (1) both species would choose the skilful actor more often than the unskilled actor, and (2) shift their initial actor’s preference before the experimental manipulation in favour of the skilful actor as this became the better option. We also examined whether the subjects engaged in a Win-stay/Lose-shift (WS/LS) strategy, i.e., basing their decisions on the outcome of the previous trial. In the two Expectation Tests, we measured subjects’ anticipatory behaviours while the two actors simultaneously manipulated a food container. Preferential looking or spatial proximity towards the skilful actor would indicate anticipation of the successful task completion and that the subjects expected the outcome of the actors’ actions. We predicted that the subjects’ anticipatory behaviours would increase toward the skilful actor while decreasing toward the unskilled actor relative to the pre-demonstration phase. We further expected this effect to strengthen from Expectation Test 1 (conducted after the first demonstration session) to Expectation Test 2 (run after both demonstrations and the Choice Test), as subjects obtained more information about the actors’ skills.

## Methods

### Subjects and study site

This study was conducted at the Centre de Primatologie – Silabe de l’Université de Strasbourg (France) between July and September 2023. Nineteen subjects participated in the study including ten Tonkean macaques (four females; 13.7 ± 5.0 years old) from two different social groups and nine brown capuchins from one social group (five females; 8.3 ± 4.4 years old; Table S1). All of them were born and raised in captivity and live in wooded outdoor enclosures with constant access to indoor rooms (more details on subjects and housing can be found in the Supplementary Materials). Individuals were fed once per day with dry pellets and once per week with fruits and vegetables, and had access to water *ad libitum*. Subjects were tested individually in experimental rooms situated next to their outdoor enclosure (Figure S1). They participated in the experiment on a voluntary basis, which may have biased our sample of subjects towards dominants, individuals who are more comfortable in the experimental rooms and/or experienced in experimental testing (see STRANGE framework; Webster and Rutz 2020). The subjects had participated in several ethological studies, but they had never been tested with social evaluation experiments before (except for one Tonkean macaque in Hirel et al. 2025a; Table S1). One brown capuchin only completed Expectation Test 1 and three trials of the Choice Test but was included in the analyses as specified below.

**Fig. 1:**
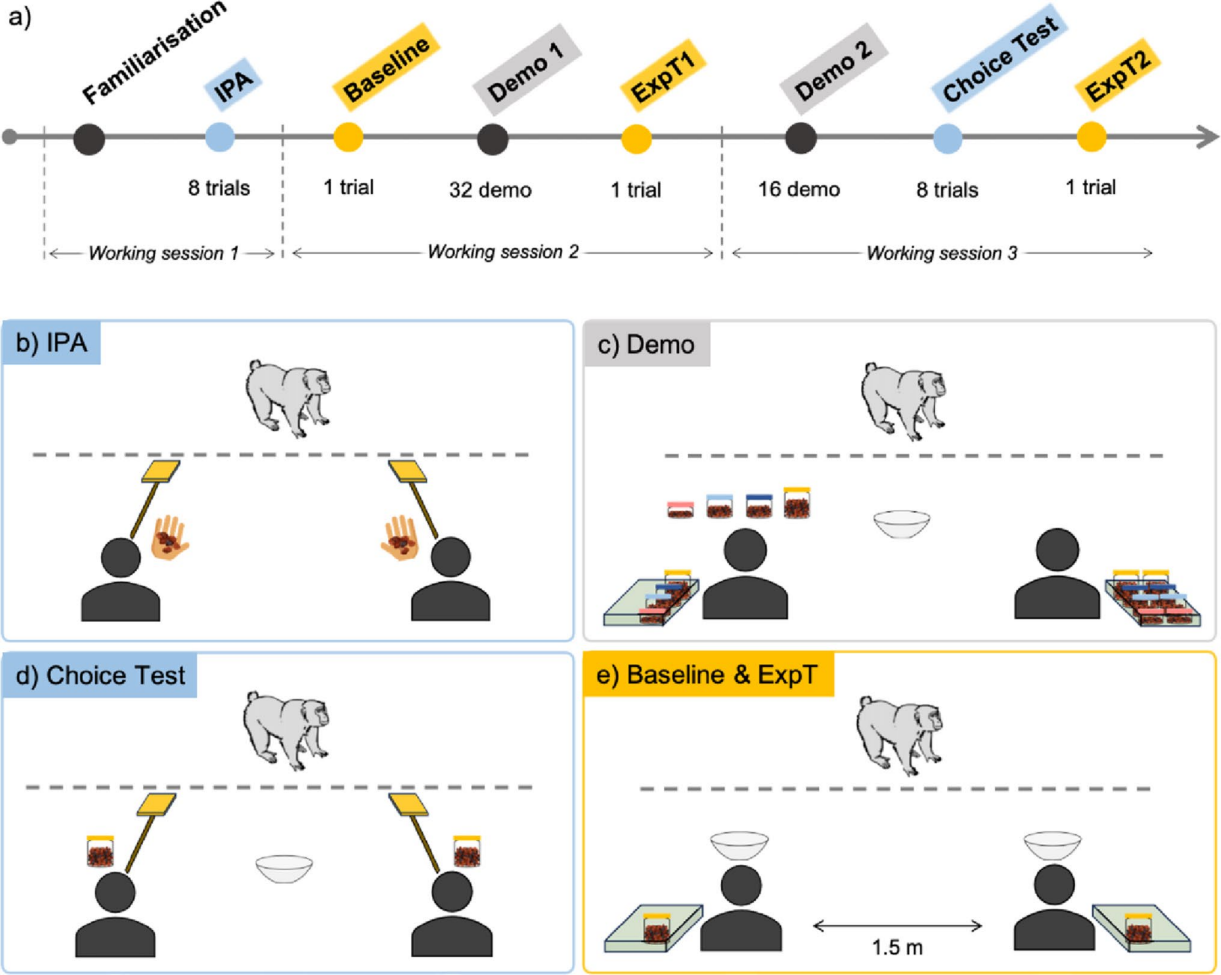
(**a**) Chronological order of the experimental steps; Materials’ and actors’ disposition at the start of (**b**) each trial of the Initial Preference Assessment (IPA): the two actors presented a target in one hand and raisins on their palm of their other hand; (**c**) the demonstration sessions: the two actors sat in front of the mesh, each with a basket containing identical sets of baited containers next to them. The bowl was in the middle, the active demonstrator had placed four containers in front of them, which they would attempt to open in a row during one demo trial. In Demo 1, the actors alternately manipulated eight containers (i.e., two demo trials) on one side before switching position to manipulate eight containers on the opposite side. In Demo 2, the actors alternately manipulated four containers on each side; (**d**) Choice Test: the two actors sat in front of the mesh and presented their target, one container was placed in front of each actor, the bowl was in the middle; (**e**) Expectation Tests (Baseline, ExpT1, ExpT2): the two actors sat in front of the mesh with their basket and one container next to them, a bowl was placed in front of each actor, each actor manipulated one container. For each step, the actors sat at the same two predetermined locations in front of the mesh of the testing room, 1.5 m apart and with a distance from the mesh adjusted for the arm length of each subject

**Table 1:** Information about the different experimental steps

Step	Type	Measure	Purpose	Analyses
Familiarisation	1 session	–	Let subjects experience themselves the possible states of the containers and how they can be opened and closed	–
Demonstration	Demo 1 (8 obs/actor), Demo 2 (4 obs/actor)	Looking time	Give subjects the opportunity to obtain information about the actors’ skills from observation	–
Initial Preference Assessment (IPA)	1 session (8 trials)	Actor choices	Assessing subjects’ initial preferences for the actors and assigning the roles of the actors	Estimation of the change in preference for the skilful actor from the IPA to the Choice Test
Choice Test	1 session (8 trials)	Actor choices	Measuring the number of choices of the subjects for the skilful actor	Estimation of the probability to choose the skilful actor according to trial number
Expectation Test	3 trials of 20 s each: Baseline, ExpT1, ExpT2	Looking & spatial proximity time	Measuring the subjects’ time of looking at and being close to the actors before the demonstrations (Baseline), after Demo 1 (ExpT1), and after both demonstrations and Choice Test (ExpT2)	Estimation of the effect of trial (Baseline, ExpT1, ExpT2) on the subjects’ time of looking at and/or in proximity to both actors

## Procedure and design

The same two familiar humans played the roles of the skilful and the unskilled actors for all subjects. The task was to open and empty transparent containers filled with raisins into a transparent bowl. The skilful actor was always successful whereas the unskilled actor always failed. The actors’ roles were constant for each subject throughout the experimental procedure and were assigned according to subjects’ choices in the Initial Preference Assessment (IPA; see below). For each experimental session, both actors were present and sat at two predetermined locations in front of the experimental room, with their locations pseudo-randomised (i.e., an equal number of times on each side) within subjects and counterbalanced between subjects (for more details on the materials, actors’ locations, and procedure explained below, please refer to the Supplementary Materials).

The experiment consisted of eight steps divided into three ‘working’ sessions for each subject (Fig. 1a; Table 1). Each subject underwent up to one working session per half day (i.e., maximum two sessions per day). To increase the possibility for the subjects to evaluate the actors’ skills at manipulating the containers, the subjects experienced the possible states of the containers (i.e., open or closed) and the outcomes in their food intake (Kuroshima et al. 2014), and how a human can open and close the containers, during a familiarisation session before the test phase. In addition, as touching the target was the measure of subjects’ choices for the actors during the IPA and Choice Test, the experimenter ensured that each subject was able to choose the better of two options by touching targets before the experiment. Only subjects who met the learning criterion (i.e., choosing the better food option on at least ten out of 12 trials in a session) could participate in the study.

### Initial preference assessment (IPA)

We conducted eight IPA trials before the experimental manipulation to assess whether subjects had a spontaneous initial preference for one of the actors, i.e., a personal preference unrelated to any experimental manipulation. First, the two actors fed the subject a few raisins, one after the other, to show the subject that both were willing to give food. Then, for each IPA trial, both actors held one raisin in the palm of their hand and a target in the other. Once the experimenter lured the subject to the central position equidistant to the actors, the actors presented their target simultaneously in front of them, and the experimenter stepped back. The subject could approach one of the actors to touch their target and obtain the food; this target touch was considered a choice for this actor (video S1). If the subjects chose each actor in four out of eight trials, the actors’ roles were randomly assigned and counterbalanced between subjects. If a subject chose one actor five times or more out of the eight trials, we assigned to this ‘preferred’ person the role of the unskilled actor for this subject. We chose this conservative role assignment rule to avoid a confound of initial preference for an actor and choice based on skill demonstrations of the actor.

### Baseline

We measured the baseline for subjects’ anticipatory behaviours before they acquired any information about the skilfulness of the actors during the demonstration sessions. The two actors had one identical closed baited container next to them and one transparent bowl in front of them (Fig. 1e). Once the experimenter lured the subject to the central position, the actors started simultaneously manipulating their container while the experimenter stepped back (video S5). After 20 s of manipulation, the skilful actor successfully opened their container and emptied it into their bowl while the unskilled actor tried in vain to empty their still closed container into their bowl. Both actors then simultaneously put their containers into their baskets and looked down. The experimenter first took the empty bowl in front of the unskilled actor to show it to the subject, then took the full bowl in front of the skilful actor to feed the subject with a few raisins. During the duration of the trial, the actors focused only on the containers and never looked at the subject.

### Demonstration sessions

Each actor alternately demonstrated their skill to open four containers in a row in one location (subsequently referred to as one demo trial). One transparent bowl was in the middle equidistant from the actors (Fig. 1c). In Demo 1 session, each actor had eight closed baited containers and alternately demonstrated twice in the same location (e.g., one skilful-left demo trial, one unskilled-right, one skilful-left, and one unskilled-right). The actors then repeated the same procedure but at the opposite location. In Demo 2 session, the procedure was the same except that the actors held four containers and only one demo trial per location was performed by each actor. We limited Demo 2 session to two demo trials so that subjects could be tested immediately afterward with the Choice Test and Expectation Test 2, while keeping the working session short enough to maintain subjects’ motivation. Therefore, the subjects observed each actor manipulating 16 containers (eight containers per side) during Demo 1 and eight containers (four containers per side) during Demo 2, resulting in a total of 24 observations per actor for each subject.

A demo trial started with an actor placing four containers aligned in front of them and then presenting their target. In the meantime, the other actor stayed head down without moving until the end of the demo trial. Once the subject had touched the target, the actor started manipulating the four containers. After trying for around five seconds, the skilful actor successfully opened and emptied each container whereas the unskilled actor failed and tried in vain to empty the closed container into the transparent bowl. After manipulating their fourth container, the actor stopped moving and looked down. The experimenter took the transparent bowl which was either filled with food (after a skilful actor’s manipulation) or empty (after an unskilled actor’s manipulation). The experimenter then fed the subject some of the food from the bowl or showed the empty bowl to the subject, before replacing the bowl with a new identical empty one (video S2). We deemed it necessary to reward the animals with small quantities of food, but not directly from the skilful actor; otherwise, they might have lost the motivation to participate in the experiment.

### Choice test

The subjects chose in eight trials which of the two actors could manipulate a container to release food. A trial began with the two actors placing one identical closed baited container in front of them and holding a target in their hand. One transparent bowl was in the central position, equidistant to the actors. Once the experimenter lured the subject to the central position, the actors presented their target simultaneously while the experimenter stepped back (Fig. 1d). The subjects indicated their choice by touching the target of one actor. The unchosen actor removed their target, put their container back, and waited with their head down until the end of the trial. Once chosen, the skilful and unskilled actors followed the same actions with their container as during the demonstration trials. The experimenter then fed the subject some of the food from the bowl (after the skilful actor’s manipulation) or showed the empty bowl to the subject (after the unskilled actor’s manipulation; videos S3 and S4). The subjects were given a maximum of 20 s to make a choice, otherwise the trial was aborted. A trial (with the same actors’ location configuration) was repeated a maximum of three times before aborting the session (which never happened).

#### Expectation test

We measured subjects’ anticipatory behaviours toward the actors in two trials, after subjects had information about the actors’ skills from Demo 1 (ExpT1) and from both demonstrations and the Choice Test (ExpT2; Fig. 1a). The procedure for ExpT1 and ExpT2 was exactly the same as for the Baseline (videos S5 and S6). The actors’ location was the same for the three trials for one subject, but counterbalanced between subjects.

## Data coding

All experimental steps were videotaped with three GoPro9 cameras to obtain different views of the subjects, the actors, setups, and experimental rooms. All the videos were coded frame by frame by an observer using Behavioral Observation Research Interactive Software (BORIS v.8.20; Friard and Gamba 2016). A second observer, who was unaware of the study design and hypothesis, coded independently 38 videos which were pseudo-randomly selected to include 20% of the sessions for each combination of phase, species, and subjects, giving a relatively representative sample of each behaviour coded. Inter-coder reliability scores ranged from 63% to 100% (see Supplementary Materials for details). For the IPA and Choice Test, videos were coded for: (a) the choices of the subjects toward either actor and (b) the synchronicity of targets’ presentation by the actors. For the Expectation Tests and the demonstration sessions, videos were coded for: (a) the duration of looking at either actor and (b) the amount of time subjects spent in close range to either actor (1 m radius), in nearby range, or far away (Figures S1 and S2). The exact definitions of the coded behaviours are reported in Table S2. Both Tonkean macaques and brown capuchins paid attention to the actors’ actions during the demonstrations around half the time (Table S3). As a measure of the subjects’ attention to both actors during the demonstration sessions that we included in the analyses, we chose the minimum looking time per subject to the skilful and unskilled actor, i.e., the time during which each subject observed each actor.

### Data analyses

#### Choices

We assessed whether subjects chose the skilful actor more often than the unskilled actor in the Choice Test and whether this probability of choosing the skilful actor increased with direct experience (i.e., trial number), by fitting a Generalized Linear Mixed model (GLMM; Baayen 2008) with logit link function (McCullagh and Nelder 1989) and a binomial error structure. The sample included 147 observations (i.e., trials) from 19 subjects (including the brown capuchin subject who completed only the first three trials of the Choice Test session). The response variable for each trial was 1 (choice of the skilful actor) or 0 (choice of the unskilled actor). The fixed-effect predictors we included in this model were trial number (main predictor), species (capuchin, Tonkean), the attention directed at the actors during the demonstration, and the three-way interaction between these predictors. This three-way interaction was included to account for the possibility that species differences, as well as individual variation in attention during the demonstration, could influence trial learning curves, and that the two species may require different amounts of time to acquire comparable levels of information about the actors’ skills. To control for their potential effects, we included the sex of the subjects, the side location (left, right), and the identity (A, B) of the skilful actor. Although we did not have a specific expectation regarding potential sex differences in social evaluation abilities, we decided to include sex as a control factor, given previous findings showing sex differences in chimpanzees and dogs (Watson et al. 2018; Chijiiwa et al. 2022). An additional control predictor, synchronicity of targets’ presentation (−1: unskilled actor first, 0: synchronous, + 1: skilful actor first), was added to the model. During video coding, we noticed that the two actors were not always fully synchronous which could have affected the choices of the subjects.

To account for individual differences, avoid overconfident model estimates, and keep type I error rate at the nominal level of 5%, we included subject ID as a random intercept effect and all identifiable random slopes within subject, which were trial number, side location of the skilful actor, and synchronicity of targets’ presentation (Schielzeth and Forstmeier 2009; Barr et al. 2013). As an overall test of the fixed effects and to avoid cryptic multiple testing (Forstmeier and Schielzeth 2011), we compared this full model with a null model lacking the effect of trial number and its interactions but being otherwise identical, using likelihood ratio tests (Dobson and Barnett 2018). To test the impact of individual fixed effects, we conducted likelihood ratio tests (Dobson and Barnett 2018) that compared the full models with reduced models, each lacking one fixed effect at a time (Barr et al. 2013). We obtained confidence intervals of model estimates and fitted values using a parametric bootstrap (*N* = 1000 bootstraps). We checked all the relevant model assumptions and transformed some variables when needed to ease the interpretation of the model estimates and convergence. This procedure of assumption checks applies to all analyses (see Supplementary Materials for more details on each model analysis).

### Shift in preference for the skilful actor

Regardless of whether the probability of choosing the skilful actor during the Choice Test differed from chance (50%), subjects might have chosen the skilful actor more often in the Choice Test than during the IPA. Therefore, we fitted a similar model except that we estimated the change in preference for the skilful actor from the IPA to the Choice Test. The sample included 288 observations (i.e., trials) from 18 subjects (excluding the brown capuchin subject who did not complete the Choice Test). The response variable was a matrix comprising the number of choices for the skilful and unskilled actors for each subject and phase. This model included the test phase (IPA, Choice Test) as the main predictor and species (capuchin, Tonkean). To account for random individual differences, avoid overconfident model estimates, and keep the type I error rate at the nominal level of 5%, we included subject ID as a random intercept effect and the only theoretically identifiable random slope phase within the subject.

### Alternative WS/LS strategy

The subjects could have used alternative strategies rather than sampling and evaluating the actors’ skills. Like chimpanzees in the study by Melis et al. (2006), they could engage in a WS/LS strategy by tracking their success with each of the actors and basing their decisions on the outcome of the previous trial. We ran a post-hoc GLMM analysis to estimate the effect of positive outcomes on the probability of staying with or switching actor choices during the Choice Test. For each trial, we coded whether the subjects stayed with or switched their choice of actors from the previous trial (variable ‘strategy’) and whether they had chosen the skilful actor and obtained a food reward in the previous trial (variable ‘reward’). The sample included seven trials per subject (trials 2 to 8), resulting in 126 trials in total. In the model, we included strategy (stay, switch) as the response variable, reward (yes, no) as a fixed effect, and species as a control predictor. We also included subject ID as a random intercept effect to account for random individual differences, to avoid overconfident model estimates, and to keep the type I error rate at the nominal level of 5%. If subjects follow the WS/LS strategy, they should base their decisions on the outcomes in the previous trial. We would observe more “stay” decisions after successes than failures and more “switch” decisions after failures than successes.

### Anticipatory behaviours

We assessed whether subjects would preferentially look or be close to the skilful actor while both actors simultaneously manipulated a baited container for 20 s. We wanted to estimate the effect of trial (Baseline, ExpT1, and ExpT2) on the subjects’ looking time and their proximity to both actors, as subjects gained more indirect and direct information about the actors’ skills. We define direct information as information obtained through direct interactions with the actors, and indirect information as information acquired solely through observation of the actors. As the response variables were bound between 0 and 20 s, we turned them into proportions to rule out fitted values and confidence intervals of fitted values extending beyond the possible response range. We fitted two GLMMs with a beta error distribution (McCullagh and Nelder 1989) and with identical structure except for the response variable, which was either the proportion of time looking at or the proportion of time spent in proximity to either actor. The sample for each model included 112 observations from 19 subjects and 56 levels of trial nested in subject (including the brown capuchin subject who completed only the Baseline and ExpT1). In these models, we included actor (skilful, unskilled), trial (Baseline, ExpT1, ExpT2) and their interaction as the main predictors. To control for their potential effects, we also included species, the sex of the subjects, and the identity of the skilful actor.

To account for random individual differences, avoid overconfident model estimates and keep the type I error rate at the nominal level of 5%, we included subject ID as a random intercept effect and all theoretically identifiable random slopes (Schielzeth and Forstmeier 2009; Barr et al. 2013), which were trial and actor within subject. In addition, we included the random intercept effect of trial nested in subject to account for the fact that the data for the skilful and unskilled actor for any given combination of subject and trial were not independent. Following the same model comparison procedure as before, we compared these full models with null models lacking the effects of trial, actor, and their interaction but being otherwise identical, and with reduced models, each lacking one fixed effect at a time, using likelihood ratio tests.

### R functions and packages

We conducted statistical analyses and created plots using R (version 4.3.2; R Core Team 2022). We used the function glmer of the package lme4 (version 1.1–35.1; Bates et al. 2015) to fit the logistic models, and the function glmmTMB of the homonymous package (version 1.1.8; Brooks et al. 2017) for the models with beta distribution. Parametric bootstraps were obtained using the functions bootMer (package lme4) and simulate (package glmmTMB).

## Results

### Choices

In the Choice Test, subjects chose the skilful actor 85 times out of 144 (brown capuchins: 39 choices, 60.9%; Tonkean macaques: 46 choices, 57.5%) and they chose the unskilled actor 59 times out of 144 (brown capuchins: 25 choices, 39%; Tonkean macaques: 34 choices, 42.5%; excluding the brown capuchin who did not complete the session). Twelve subjects (six out of nine brown capuchins and six out of ten Tonkean macaques) chose the skilful actor in the first trial (Table S1). Overall, their probability of choosing the skilful actor in the Choice Test was not influenced by any of the predictor terms (full-null model comparison: χ^2^ = 7.345, df = 4, *p* = 0.119; Table 2). This result indicates that subjects did not choose the skilful actor more often than expected by chance, nor did they increase their choices for the skilful actor over the trials.

**Table 2:** Results of the subjects’ choices full model in the Choice Test (estimates together with standard errors, 95% confidence limits, significance tests, and the estimates range obtained when dropping levels of grouping factors one at a time)

Term	Estimate	SE	CL_lower_	CL_upper_	χ^2^	df	*P*	min	max
(Intercept)	−2.745	0.999	−7.788	−1.196			^1^	−3.2	−2.269
Species	1.557	1.107	−0.73	6			^1^	0.935	1.882
Trial	2.602	1.312	0.137	9.169			^1^	2.073	3.506
Attention	−1.965	0.863	−6.074	−0.455			^1^	−2.148	−1.32
Sex	1.239	0.686	0.229	3.977	3.256	1	0.071	0.745	2.054
Side skilful	1.808	0.714	0.476	5.373	6.512	1	0.011*	1.458	2.083
Target sync	0.17	0.284	−0.539	1.073	0.362	1	0.547	0.018	0.306
ID skilful	0.188	0.72	−1.258	2.186	0.070	1	0.791	−0.25	0.577
Species × Trial	−2.148	1.689	−9.074	1.598			^1^	−3.172	−1.447
Species × Attention	2.156	1.124	−0.043	6.837			^1^	1.55	2.531
Trial × Attention	2.568	1.304	0.003	8.72			^1^	1.886	3.263
Species × Trial × Attention	−3.875	1.765	−11.708	−0.564	5.265	1	0.022*	−4.677	−2.976

Trial number was transformed to range from 0 to 1 (originally from 1 to 8). Attention and target synchronicity were z-transformed to a mean of 0 and a standard deviation of 1 (original means and standard deviations were 98.9 ± 30.56 s and 0.39 ± 0.57 s, respectively). Species, sex, side location, and identity of the skilful actor were dummy coded with brown capuchins, female, left, and actor A being their respective reference level

^1^ not indicated because of very limited interpretability

The model revealed a significant three-way interaction between trial, species, and the subjects’ attention during the demonstration phase (*p* = 0.022; Table 2 and S3, Figure S3), possibly due to multiple testing. There were no significant effects of sex (*p* = 0.071), synchronicity in target presentation (*p* = 0.547), or the identity of the skilful actor (*p* = 0.791; Table 3). Success probability was higher when the skilful actor was on the right-hand side (*p* = 0.011; Table 2). In the Choice Test, subjects chose the left side 34.7% of the time and the right side 65.3% of the time, and four subjects chose the right side in at least seven out of the eight trials. During the IPA, subjects chose the left side 30.9% of the time and the right side 69.1% of the time. These results indicate a general preference for the right side from the start of the experiment, which may have interfered with the subjects’ choices during the Choice Test.

### Shift in preference for the skilful actor

**Fig. 2:**
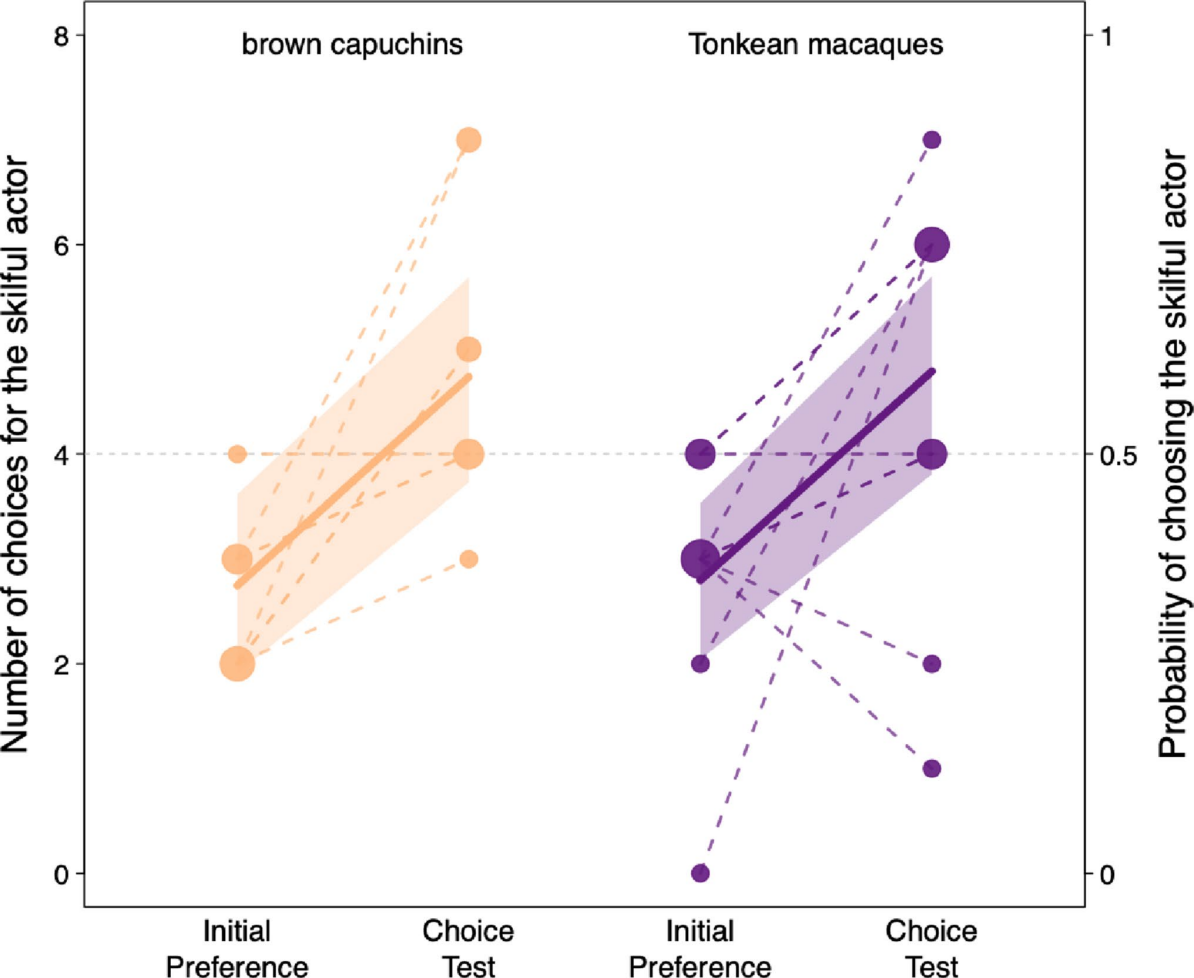
Probability of choosing the skilful actor during the Initial Preference Assessment (IPA) and the Choice Test for brown capuchins and Tonkean macaques. The continuous lines and the shaded areas depict the fitted model and its 95% confidence intervals. Points depict observations; the area of the point is proportional to the number of observations (range: 1 to 5). Observations from the same subject are connected by dashed lines

Thirteen out of the 18 subjects increased their number of choices for the skilful actor from the IPA to the Choice Test, with seven of them choosing the skilful actor in at least six out of the eight trials of the Choice Test (Table S1; Fig. 2). The model revealed a strong effect of phase (*p* < 0.001; Table 3), with the probability of choosing the skilful actor increasing from 34.7% in the IPA to 59% in the Choice Test (excluding the brown capuchin’s choices who did not complete the Choice Test; Fig. 2). The model revealed no species differences in this shift in actors’ preference (*p* = 0.917).

**Table 3:** Results of the full model for the comparison of subjects’ choices between the IPA and the Choice Test (estimates together with standard errors, 95% confidence limits, significance tests, and the estimates range obtained when dropping levels of grouping factors one at a time)

Term	Estimate	SE	CL_lower_	CL_upper_	χ^2^	df	*P*	min	max
(Intercept)	−0.648	0.23	−1.162	−0.193				−0.714	−0.571
Phase	1.021	0.286	0.482	1.591	10.228	1	0.001*	0.891	1.127
Species	0.028	0.266	−0.522	0.586	0.011	1	0.917	−0.064	0.11

The reference levels for phase and species were respectively IPA and brown capuchins

### Alternative WS/LS strategy

**Fig. 3:**
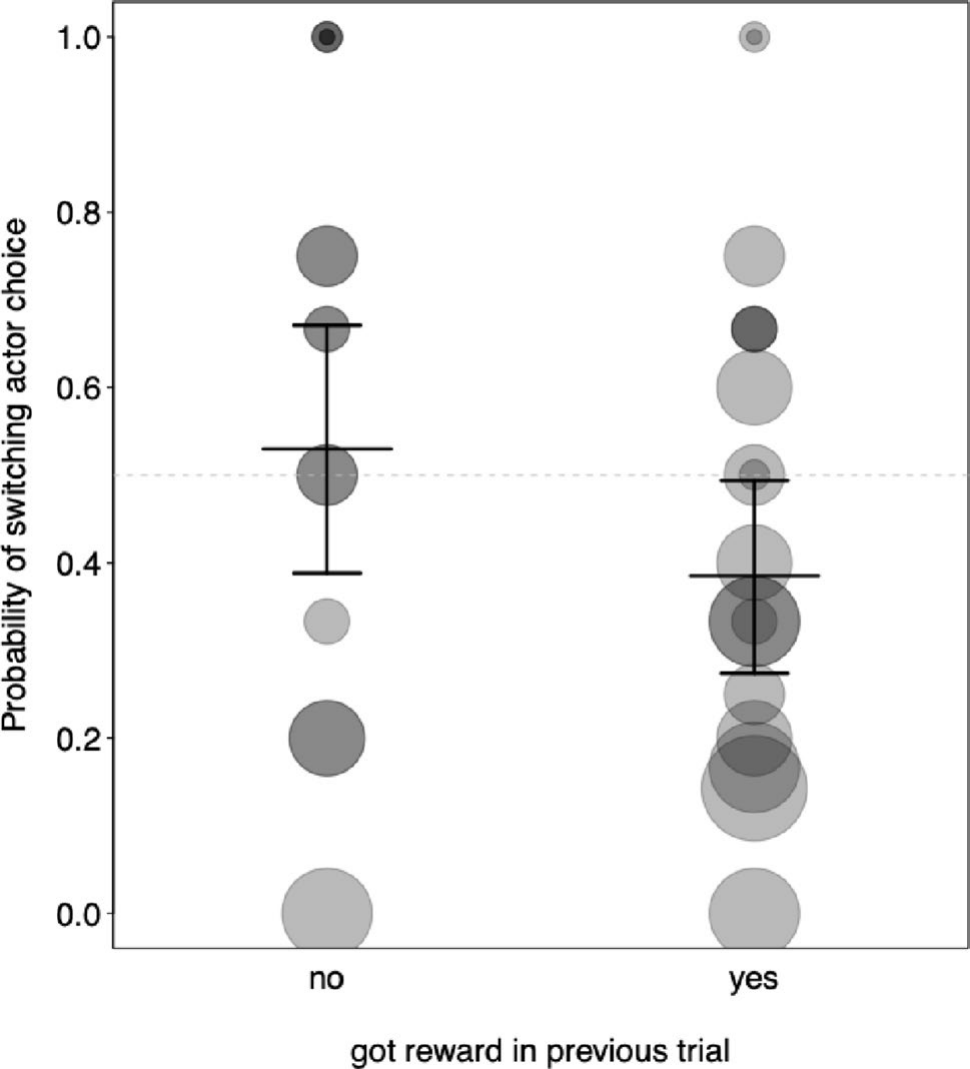
Probability of subjects switching their actor choices depending on the outcome in previous trial, i.e., whether the subjects received a reward or not. Points depict observations; the area of the point is proportional to the number of observations

After an unsuccessful trial in the Choice Test (receiving no reward), the subjects stayed with the same actor choice in 23 out of 50 trials (46%) while switched in 27 out of 50 trials (54%). After a successful trial in the Choice Test (receiving a reward), the subjects stayed with the same actor choice in 46 out of 76 trials (60.5%) while switched in 30 out of 76 trials (39.5%). The model revealed a non-significant effect of reward (*p* = 0.115) and no species differences in the probability of using the WS/LS strategy (*p* = 0.709; Table 4). Even though the subjects tended to stay with the same actor more often after a win than after a loss, they did not switch more often after a loss than after a win (Fig. 3). These results indicate that our subjects did not base their decisions on the outcomes they experienced in previous trials with a WS/LS strategy.

**Table 4:** Results of the full model for subjects’ Win-Stay/Loose-Switch (WS/LS) strategy (estimates together with standard errors, 95% confidence limits, and the estimates range)

Term	Estimate	SE	CL_lower_	CL_upper_	χ^2^	df	*P*	min	max
(Intercept)	0.198	0.356	−0.522	0.922				0.031	0.437
Reward	−0.585	0.374	−1.375	0.146	2.488	1	0.115	−0.905	−0.402
Species	−0.142	0.380	−0.898	0.686	0.140	1	0.709	−0.301	0.041

Reward was dummy coded with its reference level being no reward obtained in previous trial. The reference level for species was brown capuchins

### Anticipatory behaviours

**Fig. 4:**
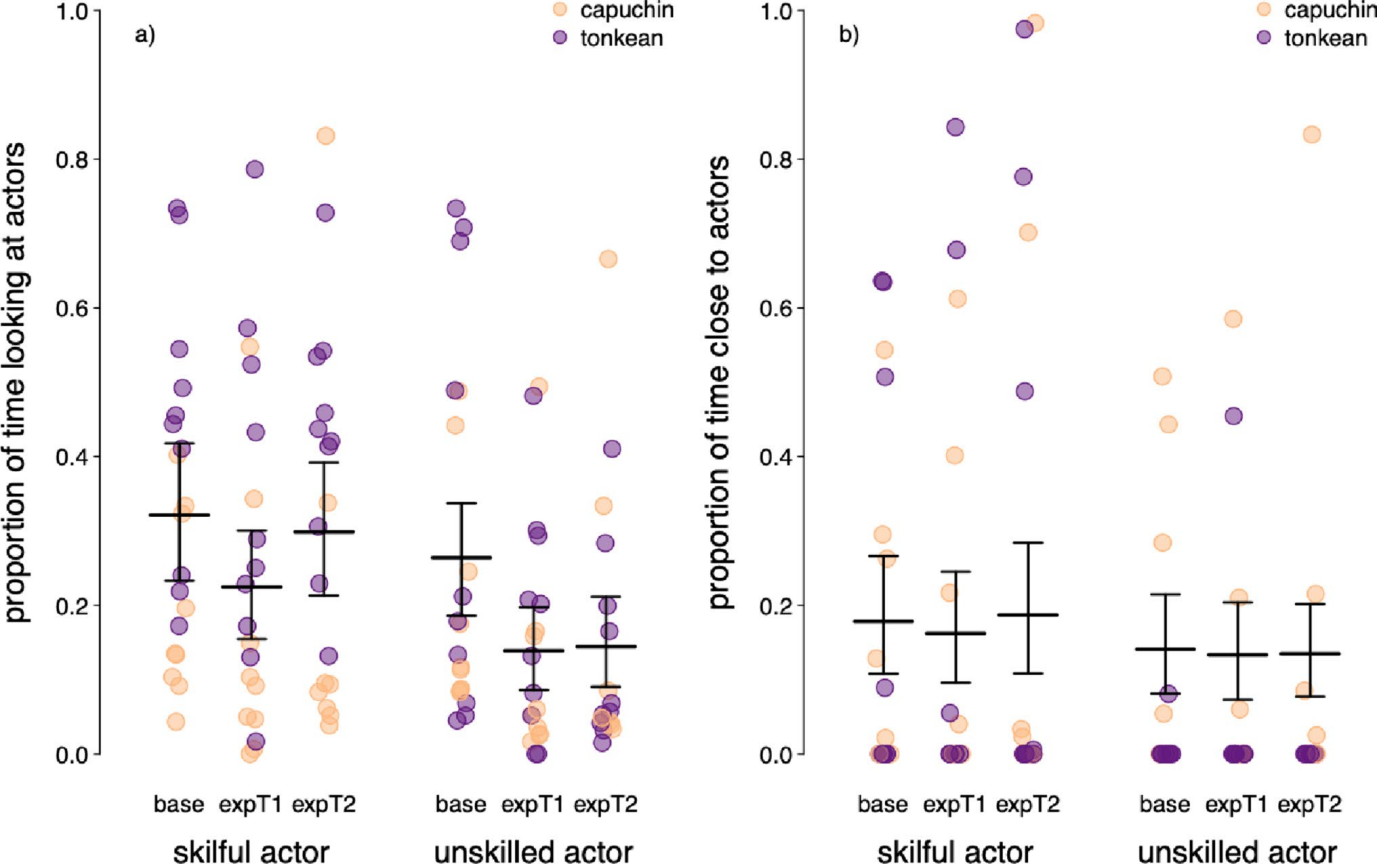
Anticipatory behaviours of the subjects during the Expectation Tests. Plot (**a**) shows the effect of the interaction between trials and actors’ roles on the proportion of time looking at the skilful and unskilled actors, while plot (**b**) shows the effect of the interaction between trials and actors’ roles on the proportion of time spent in proximity to the actors. All proportions were calculated from a total duration of 20 s. Points depict single observations (*n* = 112). Observations for brown capuchins and Tonkean macaques are shown in yellow and purple, respectively. The horizontal lines and error bars depict fitted values and their 95% confidence intervals for all non-plotted predictors being averaged. Base: Baseline; expT1: Expectation trial 1; expT2: Expectation trial 2

Overall, the proportion of time looking at the actors during the Expectation Tests was significantly influenced by trial and the actors’ role (full-null model comparison: χ^2^ = 18.5, df = 5, *p* = 0.002; Table 5). During ExpT1 and ExpT2, with all other factors being averaged, subjects looked longer at the skilful (ExpT1: 4.99 s; ExpT2: 6.44 s) than the unskilled actor (ExpT1: 2.93 s; ExpT2: 2.91 s; Fig. 4 and Table S4). They looked less at the unskilled actor during ExpT1 and ExpT2 than in the Baseline (5.42 s) but did not spend more time looking at the skilful actor than in the Baseline (6.52 s; Fig. 4 and Table S4). The model revealed significant effects of species (*p* = 0.005; Table 5 and S4; mean looking time: 12.3 s for Tonkean macaques and 6.81 s for brown capuchins), and of the skilful actor’s identity (accounting for a difference of about 1.3 s in looking time; *p* = 0.031), while the effect of sex was not significant (*p* = 0.097; Table 5 and S4).

Overall, the proportion of time spent in proximity to the actors during the Expectation Tests was not influenced by any of the predictors (full-null model comparison: χ^2^ = 2.28, df = 5, *p* = 0.808; Table S5). The proportion of time spent close to the actors was generally low and only slightly higher close to the skilful actor (ExpT1: 2.99 ± 5.44 s; ExpT2: 4.43 ± 7.47 s) than close to the unskilled actor (ExpT1: 1.38 ± 3.35 s; ExpT2: 1.29 ± 3.98 s; Table S4). However, subjects did not spend time close to each actor equally at random in the Baseline (skilful: 3.28 ± 4.78 s, unskilled: 1.44 ± 3.15 s), which may have interfered with the test results afterwards.

**Table 5:** Results of the full model for the subjects’ looking time during the Expectation Test (estimates together with standard errors, 95% confidence limits, significance tests, and the estimates range obtained when dropping levels of grouping factors one at a time)

Term	Estimate	SE	CL_lower_	CL_upper_	χ^2^	df	*P*	min	max
(Intercept)	−1.072	0.271	−1.638	−0.585			^1^	−1.204	−0.968
Phase ExpT1	−0.491	0.255	−1.023	−0.008			^1^	−0.595	−0.365
Phase ExpT2	−0.106	0.250	−0.595	0.391			^1^	−0.280	0
Actor role	−0.277	0.317	−0.941	0.310			^1^	−0.431	−0.112
Species	0.622	0.197	0.259	1.048	7.842	1	0.005**	0.508	0.821
Sex	0.329	0.199	−0.050	0.746	2.757	1	0.097	0.172	0.423
ID skilful	−0.452	0.196	−0.841	−0.067	4.660	1	0.031*	−0.614	−0.362
Phase ExpT1 × actor role	−0.309	0.365	−1.001	0.438	3.007	2	0.222	−0.448	−0.167
Phase ExpT2 × actor role	−0.650	0.369	−1.362	0.115			^2^	−0.876	−0.442

Phase, actor role, species, sex and ID of the skilful actor were dummy coded with their reference level being respectively baseline, skilful, brown capuchins, female, and actor A

^1^ not indicated because of very limited interpretability

^2^only one p-value because the interaction was tested as a whole

## Discussion

We investigated whether Tonkean macaques and brown capuchins can evaluate the skilfulness of human actors. During two demonstration sessions, subjects observed a skilful actor who consistently succeeded in opening transparent food containers and an unskilled actor who consistently failed, with rewards provided by the experimenter only following the skilful actor’s manipulations. Then, subjects completed a Choice Test and two Expectation Tests designed to assess their evaluation of the actors’ skills through different behavioural measures. When choosing which of the two actors they wanted to manipulate a container to extract food (Choice Test), subjects’ choices for the skilful actor did not differ from chance as a group. However, Tonkean macaques and brown capuchins successfully shifted their initial preference for the actors in favour of the skilful actor when this switch allowed them to maximise reward outcomes in the Choice Test. When both actors simultaneously manipulated their containers (Expectation Tests), subjects looked significantly longer at the skilful actor than the unskilled actor. They did not increase their time spent looking at the skilful actor relative to the baseline session but significantly decreased their time looking at the unskilled actor. This decrease in looking time did not become more pronounced after subjects completed the two demonstration sessions and the Choice Test (Expectation Test 2), compared with after only one demonstration session (Expectation Test 1). Unlike their looking pattern, however, subjects did not spend more time near the skilful actor than the unskilled actor, nor did their proximity patterns change across the Expectation Tests.

### Impression formation or outcome-based process?

Like other primate species (Stammbach 1988; Fruteau et al. 2009; Kendal et al. 2015; Canteloup et al. 2021; Keupp and Herrmann 2024; O’Hearn et al. 2025b; Karakoç et al. 2025), Tonkean macaques and brown capuchins in our study adapted their behaviour in relation to others’ competence levels. The experimental manipulation – i.e., the actors’ demonstrations, which subjects observed around half of the time – induced a shift in their initial choice preference toward the skilful actor and a decrease in attention to the unskilled actor. The looking pattern in the Expectation Tests is unlikely to be explained by a food-driven attention, as the two actors had the same amount of food in their containers, and we measured the subjects’ looking time during the 20 s before the skilful actor released the food. In addition, the absence of an increase in choices for the skilful actor through trials and a WS/LS strategy suggests that these monkeys did not base their decisions on the outcomes they experienced during the Choice test. These findings may reflect an ability to assess the actors’ skills based on prior observation.

Yet, our design does not allow us to disentangle impression formation from outcome-based processes. During the demonstrations, subjects received small amounts of food from the experimenter following the skilful actor’s manipulations, but not after the unskilled actor’s manipulations. This procedure was a compromise between not giving them any food at all, which would facilitate a clear interpretation of the results but considerably reduce the subjects’ level of motivation, and allowing the actors to give food. Having the experimenter, rather than the skilful actor, provide the food helped maintain the subjects’ motivation and attention, although it resulted in a different reward history following one type of demonstration. Consequently, subjects may have learned to associate the actors’ behaviours with the outcomes they experienced (i.e., receiving food or not), without necessarily forming impressions of the actors’ skills (associative learning; Heyes 2012). Social evaluation based on impression formation involves cognitive inferences – forming abstract representations of others’ characteristics and using these to infer their future behaviours (O’Hearn et al. 2025a). In addition, obtaining rewards indirectly from the experimenter may have weakened the perceived contingency between the actors’ actions and resulting outcomes, thereby making social evaluation more difficult. This aspect of the procedure may also have biased subjects’ attention during both the demonstration and test phases toward the experimenter rather than the actors. Therefore, in our study, as in previous work in which nonhuman animals experienced differential outcomes through direct interaction with others (i.e., direct information sampling; Melis et al. 2006; Carballo et al. 2015; Tan et al. 2018), the underlying mechanisms remain unclear.

### Which behavioural measures to assess social evaluation?

The discrepancies observed across our behavioural measures raise questions about which behaviours best capture social evaluation and whether all measures equally reflect it. Previous studies showed preferential attention of primates and dogs toward skilful than unskilled individuals (Ottoni et al. 2005; Tan et al. 2018; Chijiiwa et al. 2022). In our study, we observed a differential attention toward the actors characterised by a decrease in looking time at the unskilled actor. Both Tonkean macaques and brown capuchins paid less attention to the unskilled actor but did not increase attention to the skilful one. This pattern parallels the ‘negativity bias’ reported in brown capuchins, dogs, and children who showed an avoidance of antisocial over preferential choices to prosocial individuals (see Chijiiwa 2021 for a review). As in previous studies, testing choices between skilled and unskilled versus neutral actors (e.g., an actor with containers but not trying to open them) would help clarify whether this bias similarly drove our subjects’ shift in choices. However, alternative explanations for this decrease in looking time, such as habituation to the task or a decline in motivation over time, cannot be excluded. Importantly, whether social attention is a driver, a consequence of social evaluation, or both, and whether it is directly linked to social decisions, remains unclear at this point. Notably, recent large-scale research with infants evidenced that active measures, such as choice behaviour, may more reliably capture social evaluation than looking time (Lucca et al. 2025).

Our findings on choice and spatial proximity contrast with previous results. Chimpanzees preferred to approach and spent time near a prosocial than an antisocial human actor (Russell et al. 2008), and dogs show similar preferences for skilful or prosocial experimenters (Kundey et al. 2011; Marshall-Pescini et al. 2011; Carballo et al. 2015; Chijiiwa et al. 2022). While choice behaviour is the most common measure of social evaluation, results across studies are mixed. Some studies report preferences for prosocial or skilful individuals (Subiaul et al. 2008; Anderson et al. 2013b, 2016; Placì et al. 2019; Keupp and Herrmann 2024), whereas others, including ours, find that choices did not differ from chance (Kawai et al. 2019; Jim et al. 2021, 2022). Methodological factors may contribute to this variability. The number of choice trials, for instance, ranges widely across studies (e.g., 8 in ours vs. 144 trials in Anderson et al. 2013b, 2016). Our subjects also exhibited a right-side bias from the start of the experiment, which may have interfered with their choice probability. This bias could reflect the testing environment (e.g., the trapdoor to access the experimental room from the park of the brown capuchins was on the right side) or reduced interest in the task. Although given that the side of the successful choice alternated pseudo-randomly within sessions (i.e., an equal number of times on each side), a side bias represents a very efficient and less cognitively demanding strategy to obtain half of the rewards.

In addition, the social agents to evaluate are sometimes conspecifics, sometimes humans – with both significant and null effects reported. Fully controlled conspecific-based paradigms are methodologically challenging, e.g., controlling pre-existing social relationships, standardising demonstrations, and counterbalancing roles. Alternatively, testing nonhuman animals with human actors may reduce social relevance and their engagement in the task. Evaluating heterospecifics may also require distinct or more demanding cognitive processes, limiting interpretations of social evaluation. Spatial proximity measure may be particularly irrelevant in this case and not a reliable proxy for social expectation. Furthermore, several studies lacked controls for initial preferences and may have then wrongly assumed that subjects’ initial choices before the sampling phase were at chance level – particularly with demonstrators familiar to the subjects. Initial preference assessment and baseline tests also allow very informative and direct measurement of preference and behavioural change – an approach potentially more comparable to observed behavioural shifts toward skilled conspecifics in wild populations (O’Hearn et al. 2025b; Karakoç et al. 2025).

Finally, the extent to which nonhuman animals use evaluations of others’ characteristics to guide social decisions remains unclear. Social evaluation could inform both active choices (e.g., interaction, approach, or avoidance) and more indirect behaviours (e.g., observation, spatial proximity). However, across species, social evaluation may guide behaviours depending on the species-specific relevance of the context and the trait being assessed. For instance, successful cooperation can require selecting partners based on perceived willingness to cooperate, competence, or prosociality (Wu et al. 2016; Manrique et al. 2021) and social learning can benefit from merely observing competent or knowledgeable models (Laland 2004; Camacho-Alpízar and Guillette 2023). Our study context and task, which did not involve learning or cooperation with conspecifics, did affect subjects’ anticipatory-looking but not their active choices for the actors. Whether this difference in our behavioural measures reflects a context-specific effect on behavioural display of social evaluation warrants further investigation.

### Species differences in social evaluation

During both the Demonstration and Expectation Test sessions, Tonkean macaques generally looked longer at the actors than the brown capuchins. Interspecific differences in preferential choices for prosocial over antisocial agents have also been reported previously (Russell et al. 2008; Herrmann et al. 2013; Kawai et al. 2019). Although we did not predict differences between the two species we tested, variation in how species acquire and use social information may explain these results and raises questions about the relevance of behavioural measures across taxa. Strategies for social information acquisition and use likely vary between species with different social organisations (e.g., Scheid et al. 2007; Range et al. 2009; Faraut and Fischer 2019). In large groups, direct information sampling can be time-consuming – interacting with each group member requires a lot of time that is not devoted to other activities – while indirect information sampling (e.g., looking, hearing) may be more efficient or safer for less tolerant species, as direct interactions can be associated with a high risk of getting injured. These social factors can similarly affect how species use their social evaluation (e.g., by preferentially observing rather than directly interacting with others). However, since both Tonkean macaques and brown capuchins live in similar group sizes and are characterised as socially tolerant species (Fragaszy et al. 2004; Thierry et al. 2004), group size or tolerance alone are unlikely to account for the observed differences in looking behaviour.

Differences in cognitive processing may offer an alternative explanation, with some species or individuals picking up on informational cues faster than others. The amount of social information needed to form impressions of others is unknown. In our study, looking time toward the unskilled actor decreased after a single demonstration session (32 container manipulations, 16 per actor), whereas no choice preference for the skilful actor (but a shift in preference) was observed. Notably, some species – including Tonkean macaques – did not demonstrate the use of social information about others’ skills or prosociality without direct experience (Nitzschner et al. 2012; Jim et al. 2021; Hirel et al. 2025a, b), whereas chimpanzees and orangutans did (Russell et al. 2008; Subiaul et al. 2008; Herrmann et al. 2013; Keupp and Herrmann 2024). These findings suggest possible species differences in social evaluation abilities, underlying cognitive mechanisms, or strategies for acquiring social information.

## Conclusion

Tonkean macaques and brown capuchins did not choose the skilful actor more often than the unskilled actor, but they successfully shifted their initial preference in favour of the skilful actor. They neither learned through choice trials nor based their choices on previous trials outcomes. Both species looked more at the skilful over the unskilled actor when both actors simultaneously manipulated a container, seemingly due to a decrease in attention to the unskilled actor, but did not change their time spent near either actor. These results suggest that these monkeys may have used social information about the actors’ actions acquired from the demonstration sessions to adapt their behaviour. Yet, because subjects experienced differential rewarding during demonstrations, it remains unclear whether they formed impressions of the actors’ skills or based their decisions on previous outcomes. The mechanisms underlying looking behaviour and the measures that most accurately reflect social evaluation in nonhuman animals also deserve to be clarified. We encourage further research across diverse taxa to explore the cognitive processes underlying social information acquisition in nonhuman animals and the social environments that promote its emergence.

The methodological conundrum in balancing the absence of rewards with maintaining subjects’ motivation, which we faced in this study, highlights one of the challenges of investigating social evaluation in nonhuman animals, as sometimes it is not possible to rely exclusively on indirect information sampling to seed the experimental manipulations. Future studies should also carefully consider methodological design, for instance by comparing direct versus indirect information sampling, using conspecifics rather than human actors, controlling for initial preferences or behavioural baselines, and testing across multiple social contexts (e.g., cooperation, competition, social learning) using different behavioural measures (e.g., choice, looking, proximity). The approaches developed by Hirel et al. (2025a) and Keupp and Herrmann (2024) in Tonkean macaques and chimpanzees offer promising paradigms in this regard, adaptable to different species and contexts.

## Supplementary Information

Below is the link to the electronic supplementary material.

Supplementary Material 1

## Data Availability

The supplementary document, videos, data, and statistical code associated with this article can be found at: https://osf.io/gm7q4/.

## References

[CR1] Anderson JR (1985) Development of tool-use to obtain food in a captive group of *Macaca tonkeana*. J Hum Evol 14:637–645. 10.1016/S0047-2484(85)80072-5

[CR2] Anderson JR, Kuroshima H, Takimoto A, Fujita K (2013a) Third-party social evaluation of humans by monkeys. Nat Commun 4:1561. 10.1038/ncomms2495 23463004 10.1038/ncomms2495

[CR3] Anderson JR, Takimoto A, Kuroshima H, Fujita K (2013b) Capuchin monkeys judge third-party reciprocity. Cognition 127:140–146. 10.1016/j.cognition.2012.12.007 23376298 10.1016/j.cognition.2012.12.007

[CR4] Anderson JR, Bucher B, Kuroshima H, Fujita K (2016) Evaluation of third-party reciprocity by squirrel monkeys (*Saimiri sciureus*) and the question of mechanisms. Anim Cogn 19:813–818. 10.1007/s10071-016-0980-7 27021433 10.1007/s10071-016-0980-7

[CR6] Baayen RH (2008) Analyzing Linguistic Data. Cambridge University Press, Cambridge, UK

[CR7] Barr DJ, Levy R, Scheepers C, Tily HJ (2013) Random effects structure for confirmatory hypothesis testing: keep it maximal. J Mem Lang 68:255–278. 10.1016/j.jml.2012.11.001 10.1016/j.jml.2012.11.001 PMC3881361 24403724

[CR8] Bates D, Mächler M, Bolker B, Walker S (2015) Fitting linear mixed-effects models using lme4. J Stat Softw 67:1–48. 10.48550/arXiv.1406.5823

[CR9] Brooks ME, Kristensen K, van Benthem KJ et al (2017) GlmmTMB balances speed and flexibility among packages for zero-inflated generalized linear mixed modeling. R J 9:378–400. 10.3929/ethz-b-000240890

[CR10] Bshary R, Grutter AS (2006) Image scoring and cooperation in a cleaner fish mutualism. Nature 441:975–978. 10.1038/nature04755 16791194 10.1038/nature04755

[CR11] Camacho-Alpízar A, Guillette LM (2023) From whom do animals learn? A meta-analysis on model-based social learning. Psychon Bull Rev 30:863–881. 10.3758/s13423-022-02236-4 36609963 10.3758/s13423-022-02236-4

[CR12] Canteloup C, Meunier H (2017) ‘Unwilling’ versus ‘unable’: Tonkean macaques’ understanding of human goal-directed actions. PeerJ 5:e3227. 10.7717/peerj.3227 28480137 10.7717/peerj.3227 PMC5419206

[CR13] Canteloup C, Piraux E, Poulin N, Meunier H (2016) Do Tonkean macaques (*Macaca tonkeana*) perceive what conspecifics do and do not see? PeerJ 4:e1693. 10.7717/peerj.1693 26925323 10.7717/peerj.1693 PMC4768696

[CR14] Canteloup C, Poitrasson I, Anderson JR et al (2017) Factors influencing deceptive behaviours in Tonkean macaques (*Macaca tonkeana*). Behaviour 154:765–784. 10.1163/1568539X-00003443

[CR15] Canteloup C, Cera MB, Barrett BJ, van de Waal E (2021) Processing of novel food reveals payoff and rank-biased social learning in a wild primate. Sci Rep 11:9550. 10.1038/s41598-021-88857-6 34006940 10.1038/s41598-021-88857-6 PMC8131368

[CR16] Carballo F, Freidin E, Putrino N et al (2015) Dog’s discrimination of human selfish and generous attitudes: the role of individual recognition, experience, and experimenters’ gender. PLoS ONE 10:e0116314. 10.1371/journal.pone.0116314 25714915 10.1371/journal.pone.0116314 PMC4340621

[CR17] Chijiiwa H (2021) Social Evaluation in Non-human Animals. In: Anderson JR, Kuroshima H (eds) Comparative Cognition: Commonalities and Diversity. Springer, Singapore, pp 221–232

[CR18] Chijiiwa H, Horisaki E, Hori Y et al (2022) Female dogs evaluate levels of competence in humans. Behav Processes 203:104753. 10.1016/j.beproc.2022.104753 36179930 10.1016/j.beproc.2022.104753

[CR19] Corriveau KH, Meints K, Harris PL (2009) Early tracking of informant accuracy and inaccuracy. Br J Dev Psychol 27:331–342. 10.1348/026151008X310229 19998535 10.1348/026151008x310229

[CR20] De Waal FBM (2000) Attitudinal reciprocity in food sharing among brown capuchin monkeys. Anim Behav 60:253–261. 10.1006/anbe.2000.1471 10973728 10.1006/anbe.2000.1471

[CR21] Defolie C, Malassis R, Serre M, Meunier H (2015) Tufted capuchins (*Cebus apella*) adapt their communicative behaviour to human’s attentional states. Anim Cogn 18:747–755. 10.1007/s10071-015-0841-9 25630371 10.1007/s10071-015-0841-9

[CR22] Dobson AJ, Barnett AG (2018) An introduction to generalized linear models. Chapman & Hall/CRC, Boca Raton, FL

[CR23] Ducoing AM, Thierry B (2005) Tool-use learning in Tonkean macaques (*Macaca tonkeana*). Anim Cogn 8:103–113. 10.1007/s10071-004-0240-0 15449102 10.1007/s10071-004-0240-0

[CR24] Faraut L, Fischer J (2019) How life in a tolerant society affects the attention to social information in baboons. Anim Behav 152:11–17. 10.1016/j.anbehav.2019.04.004

[CR26] Forstmeier W, Schielzeth H (2011) Cryptic multiple hypotheses testing in linear models: overestimated effect sizes and the winner’s curse. Behav Ecol Sociobiol 65:47–55. 10.1007/s00265-010-1038-5 21297852 10.1007/s00265-010-1038-5 PMC3015194

[CR27] Fragaszy DM, Visalberghi E, Fedigan LM (2004) The Complete Capuchin: The Biology of the Genus Cebus. Cambridge University Press

[CR28] Friard O, Gamba M (2016) BORIS: a free, versatile open-source event-logging software for video/audio coding and live observations. Methods Ecol Evol 7:1325–1330. 10.1111/2041-210X.12584

[CR29] Fruteau C, Voelkl B, van Damme E, Noë R (2009) Supply and demand determine the market value of food providers in wild vervet monkeys. Proc Natl Acad Sci U S A 106:12007–12012. 10.1073/pnas.0812280106 10.1073/pnas.0812280106 PMC2706267 19581578

[CR30] Fusaro M, Corriveau KH, Harris PL (2011) The good, the strong, and the accurate: preschoolers’ evaluations of informant attributes. J Exp Child Psychol 110:561–574. 10.1016/j.jecp.2011.06.008 21802693 10.1016/j.jecp.2011.06.008

[CR31] Hermes J, Behne T, Studte K et al (2016) Selective cooperation in early childhood – how to choose models and partners. PLoS One 11:e0160881. 10.1371/journal.pone.0160881 27505043 10.1371/journal.pone.0160881 PMC4978381

[CR32] Hermes J, Behne T, Bich AE et al (2017) Children’s selective trust decisions: rational competence and limiting performance factors. Dev Sci 21:e12527. 10.1111/desc.12527 10.1111/desc.12527 28229561

[CR33] Herrmann E, Keupp S, Hare B et al (2013) Direct and indirect reputation formation in nonhuman great apes (*Pan paniscus*, *Pan troglodytes*, *Gorilla gorilla*, *Pongo pygmaeus*) and human children (*Homo sapiens*). J Comp Psychol 127:63–75. 10.1037/a0028929 22746158 10.1037/a0028929

[CR34] Heyes C (2012) Simple minds: a qualified defence of associative learning. Philos Trans R Soc Lond B Biol Sci 367:2695–2703. 10.1098/rstb.2012.0217 22927568 10.1098/rstb.2012.0217 PMC3427553

[CR35] Hirel M, Meunier H, Mundry R et al (2025a) Choose your partner: social evaluation of skillfulness at cooperative co-action tasks in Tonkean macaques (*Macaca tonkeana*). Anim Behav Cogn 12:330–359. 10.26451/abc.12.03.02.2025

[CR36] Hirel M, Meunier H, Rakoczy H et al (2025b) Tonkean macaques do not prefer the helper or the hinderer in the hill paradigm. R Soc Open Sci 12:250488. 10.1098/rsos.250488 40881987 10.1098/rsos.250488 PMC12381498

[CR37] Horner V, Proctor D, Bonnie KE et al (2010) Prestige affects cultural learning in chimpanzees. PLoS One 5:e10625. 10.1371/journal.pone.0010625 20502702 10.1371/journal.pone.0010625 PMC2873264

[CR38] Jim H-L, Range F, Marshall-Pescini S et al (2021) Investigating indirect and direct reputation formation in Asian elephants (*Elephas maximus*). Front Psychol. 10.3389/fpsyg.2020.604372 33519611 10.3389/fpsyg.2020.604372 PMC7841644

[CR39] Jim H-L, Plohovich M, Marshall-Pescini S, Range F (2022) Wolves and dogs fail to form reputations of humans after indirect and direct experience in a food-giving situation. PLoS One 17:e0271590. 10.1371/journal.pone.0271590 35976865 10.1371/journal.pone.0271590 PMC9385025

[CR40] Karakoç E, Vogg R, Marziliano M et al (2025) Foraging competence and its impact on social relationships in a socially tolerant wild primate. Anim Cogn 28:86. 10.1007/s10071-025-02011-7 41160154 10.1007/s10071-025-02011-7 PMC12572059

[CR41] Kawai N, Yasue M, Banno T, Ichinohe N (2014) Marmoset monkeys evaluate third-party reciprocity. Biol Lett 10:20140058. 10.1098/rsbl.2014.0058 24850892 10.1098/rsbl.2014.0058 PMC4046368

[CR42] Kawai N, Nakagami A, Yasue M et al (2019) Common marmosets (*Callithrix jacchus*) evaluate third-party social interactions of human actors but Japanese monkeys (*Macaca fuscata*) do not. J Comp Psychol 133:488–495. 10.1037/com0000182 31021114 10.1037/com0000182

[CR43] Kendal R, Hopper LM, Whiten A et al (2015) Chimpanzees copy dominant and knowledgeable individuals: implications for cultural diversity. Evol Hum Behav 36:65–72. 10.1016/j.evolhumbehav.2014.09.002 27053916 10.1016/j.evolhumbehav.2014.09.002 PMC4820294

[CR44] Keupp S, Herrmann E (2024) Domain-specific inferences about conspecifics’ skills by chimpanzees. Sci Rep 14:21996. 10.1038/s41598-024-73340-9 39313494 10.1038/s41598-024-73340-9 PMC11420200

[CR45] Kundey SMA, De Los Reyes A, Royer E et al (2011) Reputation-like inference in domestic dogs (*Canis familiaris*). Anim Cogn 14:291–302. 10.1007/s10071-010-0362-5 21140184 10.1007/s10071-010-0362-5

[CR46] Kuroshima H, Kaiser I, Fragaszy DM (2014) Does own experience affect perception of others’ actions in capuchin monkeys (*Cebus apella*)? Anim Cogn 17:1269–1279. 10.1007/s10071-014-0760-1 24844666 10.1007/s10071-014-0760-1

[CR47] Kushnir T, Vredenburgh C, Schneider LA (2013) Who can help me fix this toy? The distinction between causal knowledge and word knowledge guides preschoolers’ selective requests for information. Dev Psychol 49:446–453. 10.1037/a0031649 23339590 10.1037/a0031649

[CR48] Laland KN (2004) Social learning strategies. Anim Learn Behav 32:4–14. 10.3758/BF03196002 10.3758/bf03196002 15161136

[CR49] Leete J, Vonk J, Oriani S et al (2020) Do domestic cats (*Felis silvestris catus*) infer reputation in humans after direct and indirect experience? Hum-Anim Interact Bull 8:35–53. 10.1079/hai.2020.0016

[CR50] Lucca K, Yuen F, Wang Y et al (2025) Infants’ social evaluation of helpers and hinderers: a large-scale, multi-lab, coordinated replication study. Dev Sci 28:e13581. 10.1111/desc.13581 39600132 10.1111/desc.13581

[CR52] Manrique HM, Zeidler H, Roberts G et al (2021) The psychological foundations of reputation-based cooperation. Philos Trans R Soc Lond B Biol Sci 376:20200287. 10.1098/rstb.2020.0287 34601920 10.1098/rstb.2020.0287 PMC8487732

[CR53] Marshall-Pescini S, Passalacqua C, Ferrario A et al (2011) Social eavesdropping in the domestic dog. Anim Behav 81:1177–1183. 10.1016/j.anbehav.2011.02.029

[CR54] McCullagh P, Nelder JA (1989) Generalized linear models, 2nd edn. Chapman & Hall, London

[CR55] Melis AP, Hare B, Tomasello M (2006) Chimpanzees recruit the best collaborators. Science 311:1297–1300. 10.1126/science.1123007 16513985 10.1126/science.1123007

[CR56] Nitzschner M, Melis AP, Kaminski J, Tomasello M (2012) Dogs (*Canis familiaris*) evaluate humans on the basis of direct experiences only. PLoS One 7:e46880. 10.1371/journal.pone.0046880 23056507 10.1371/journal.pone.0046880 PMC3466196

[CR82] O'HearnW, Hirel M, Keupp S, Fischer J (2025a) Social evaluation of skill and competence in primates. Neurosci Biobehav Rev. 177:106346. 10.1016/j.neubiorev.2025.106346 40845934 10.1016/j.neubiorev.2025.106346

[CR57] O’Hearn WJ, Beckmann J, Von Fersen L et al (2025b) Increased female competition for males with enhanced foraging skills in Guinea baboons. Proc R Soc B Biol Sci 292:20242925. 10.1098/rspb.2024.2925 10.1098/rspb.2024.2925 PMC11880840 40040456

[CR58] Ottoni EB, Izar P (2008) Capuchin monkey tool use: overview and implications. Evol Anthropol Issues News Rev 17:171–178. 10.1002/evan.20185

[CR59] Ottoni EB, de Resende BD, Izar P (2005) Watching the best nutcrackers: what capuchin monkeys (*Cebus apella*) know about others’ tool-using skills. Anim Cogn 8:215–219. 10.1007/s10071-004-0245-8 15719240 10.1007/s10071-004-0245-8

[CR60] Phillips W, Barnes JL, Mahajan N et al (2009) ‘Unwilling’ versus ‘unable’: capuchin monkeys’ (*Cebus apella*) understanding of human intentional action. Dev Sci 12:938–945. 10.1111/j.1467-7687.2009.00840.x 19840049 10.1111/j.1467-7687.2009.00840.x

[CR61] Piotti P, Spooner RM, Jim H-L, Kaminski J (2017) Who to ask for help? Do dogs form an opinion on humans based on skilfulness? Appl Anim Behav Sci 195:93–102. 10.1016/j.applanim.2017.05.024

[CR62] Placì S, Padberg M, Rakoczy H, Fischer J (2019) Long-tailed macaques extract statistical information from repeated types of events to make rational decisions under uncertainty. Sci Rep 9:12107. 10.1038/s41598-019-48543-0 31431638 10.1038/s41598-019-48543-0 PMC6702217

[CR63] R Core Team (2022) R: A language and environment for statistical computing. R Found Stat Comput Vienna Austria

[CR64] Rakoczy H, Warneken F, Tomasello M (2009) Young children’s selective learning of rule games from reliable and unreliable models. Cogn Dev 24:61–69. 10.1016/j.cogdev.2008.07.004

[CR65] Range F, Horn L, Bugnyar T et al (2009) Social attention in keas, dogs, and human children. Anim Cogn 12:181–192. 10.1007/s10071-008-0181-0 18716802 10.1007/s10071-008-0181-0 PMC4415148

[CR66] Riley EP (2010) The endemic seven: four decades of research on the Sulawesi macaques. Evol Anthropol Issues News Rev 19:22–36. 10.1002/evan.20246

[CR67] Russell YI, Call J, Dunbar RIM (2008) Image scoring in great apes. Behav Processes 78:108–111. 10.1016/j.beproc.2007.10.009 18068313 10.1016/j.beproc.2007.10.009

[CR68] Scheid C, Range F, Bugnyar T (2007) When, what, and whom to watch? Quantifying attention in Ravens (*Corvus corax*) and jackdaws (*Corvus monedula*). J Comp Psychol 121:380–386. 10.1037/0735-7036.121.4.380 18085921 10.1037/0735-7036.121.4.380

[CR69] Schielzeth H, Forstmeier W (2009) Conclusions beyond support: overconfident estimates in mixed models. Behav Ecol 20:416–420. 10.1093/beheco/arn145 19461866 10.1093/beheco/arn145 PMC2657178

[CR70] Sih A, Sinn DL, Patricelli GL (2019) On the importance of individual differences in behavioural skill. Anim Behav 155:307–317. 10.1016/j.anbehav.2019.06.017

[CR71] Stammbach E (1988) Group responses to specially skilled individuals in a *Macaca fascicularis* group. Behav 107:241–266. 10.1163/156853988X00368

[CR72] Subiaul F, Vonk J, Okamoto-Barth S, Barth J (2008) Do chimpanzees learn reputation by observation? Evidence from direct and indirect experience with generous and selfish strangers. Anim Cogn 11:611–623. 10.1007/s10071-008-0151-6 18357476 10.1007/s10071-008-0151-6

[CR73] Tan A, Hemelrijk C, Malaivijitnond S, Gumert M (2018) Young macaques (*Macaca fascicularis*) preferentially bias attention towards closer, older, and better tool users. Anim Cogn 21:551–563. 10.1007/s10071-018-1188-9 29754253 10.1007/s10071-018-1188-9

[CR74] Thierry B (2007) Unity in diversity: lessons from macaque societies. Evol Anthropol Issues News Rev 16:224–238. 10.1002/evan.20147

[CR75] Thierry B, Anderson JR, Demaria C et al (1994) Tonkean macaque behaviour from the perspective of the evolution of Sulawesi macaques. In: Roeder JJ, Thierry B, Anderson JR, Herrenschmidt N (eds) Current Primatology, Vol.2: social development, learning and behaviour. Université Louis Pasteur, Strasbourg, pp 103–117

[CR76] Thierry B, Singh M, Kaumanns W (2004) Macaque societies: A model for the study of social organization. Cambridge University Press, Cambridge, UK

[CR77] Vail AL, Manica A, Bshary R (2014) Fish choose appropriately when and with whom to collaborate. Curr Biol 24:R791–R793. 10.1016/j.cub.2014.07.033 25202866 10.1016/j.cub.2014.07.033

[CR78] Watson SK, Vale GL, Hopper LM et al (2018) Chimpanzees demonstrate individual differences in social information use. Anim Cogn 21:639–650. 10.1007/s10071-018-1198-7 29922865 10.1007/s10071-018-1198-7 PMC6097074

[CR79] Webster MM, Rutz C (2020) How STRANGE are your study animals? Nature 582:337–340. 10.1038/d41586-020-01751-5 32541916 10.1038/d41586-020-01751-5

[CR80] Whitehouse J, Meunier H (2020) An understanding of third-party friendships in a tolerant macaque. Sci Rep 10:9777. 10.1038/s41598-020-66407-w 32555440 10.1038/s41598-020-66407-w PMC7300006

[CR81] Wu J, Balliet D, Van Lange PAM (2016) Reputation, gossip, and human cooperation. Soc Personal Psychol Compass 10:350–364. 10.1111/spc3.12255

