# Supplementary material for: Social evaluation of skilfulness in Tonkean macaques (Macaca tonkeana) and brown capuchins (Sapajus apella)
Source: Anim Cogn. 2026 Jan 14;29(1):15. doi: 10.1007/s10071-025-02039-9 (PMC12819564; doi:10.1007/s10071-025-02039-9)
Supplement: Supplementary file 1 — Supplementary Material 1 [file 10071_2025_2039_MOESM1_ESM.docx]

Supplementary Materials

Social evaluation of skilfulness in Tonkean macaques (*Macaca tonkeana*) and brown capuchins (*Sapajus apella*)

Marie Hirel^1,2^, Michele Marziliano^1,2^, Hélène Meunier^4,5^, Hannes Rakoczy^3,6^, Julia Fischer^1,2,3^, Stefanie Keupp^1,2,3^

^1^ Georg-August-Universität Göttingen, Johann-Friedrich-Blumenbach Institute, Department for Primate Cognition, Kellnerweg 4, 37077 Göttingen, Germany

^2^ German Primate Center – Leibniz Institute for Primate Research, Cognitive Ethology Laboratory, Kellnerweg 4, 37077 Göttingen, Germany

^3^ Leibniz ScienceCampus, German Primate Center – Leibniz Institute for Primate Research, Kellnerweg 4, 37077 Göttingen, Germany

^4^ Centre de Primatologie de l’Université de Strasbourg, Chemin du Fort Foch, 67207 Niederhausbergen, France

^5^ Laboratoire de Neurosciences Cognitives et Adaptatives, UMR 7364, 12 rue Goethe, 67000 Strasbourg, France

^6^ Georg-August-Universität Göttingen, Georg-Elias-Müller Institute for Psychology, Department for Cognitive Developmental Psychology, Waldweg 26, 37073 Göttingen, Germany

## Supplemental videos S1-S6

All six videos can be found at: <https://osf.io/gm7q4/>

## Methods

### Subjects and living conditions

The 19 subjects who participated in the study lived in three different social groups (Table S1). Group 1 was composed of 29 Tonkean macaques (19 females) aged four months to 27 years (mean age: 7.6 years), living in a wooded outdoor enclosure of 3788 m^2^ with constant access to an indoor room of 20 m^2^. Group 2 was composed of six male Tonkean macaques aged 12 to 16 years (mean age: 14.7 years), living in a wooded outdoor enclosure of 1364 m^2^ with constant access to an indoor room of 10 m^2^. Group 3 was composed of 18 brown capuchins (11 females) aged four months to 15 years (mean age: 6.8 years), living in a wooded outdoor enclosure of 2332 m^2^ with constant access to an indoor room of 16.2 m^2^.

### Schematic of the experimental rooms

The experiment was carried out individually in experimental rooms of about 12 m^2^ situated next to the groups’ outdoor enclosures (see Figure S1). Subjects participated in the experiment on a voluntary basis. They were temporarily separated from their group in the experimental room if they were comfortable with it; otherwise, the sliding door to access the experimental room from their park was kept open, and subjects were tested only when the other group members were not in the surroundings.

| Table S1: Overview of subjects and their choices in the Initial Preference Assessment (IPA) and the Choice Test. | | | | | | | | |
| --- | --- | --- | --- | --- | --- | --- | --- | --- |
| **Subject** | **Species** | **Social group** | **Sex** | **Age (years)** | **Skilful**  **actor** | **IPA**  **(# skilful)** | **Choice test**  **(# skilful)** | **First trial choice** |
| Dory | Tonkean | 1 | F | 7 | A | 3/8 | 2/8 | skilful |
| Eric | Tonkean | 1 | M | 6 | A | 3/8 | 1/8 | skilful |
| Nema | Tonkean | 1 | F | 12 | A | 4/8 | 6/8 | skilful |
| Nereis^1^ | Tonkean | 1 | F | 24 | B | 0/8 | 6/8 | skilful |
| Patchouli | Tonkean | 1 | M | 12 | B | 3/8 | 7/8 | unskilled |
| Yin | Tonkean | 1 | F | 14 | A | 4/8 | 4/8 | unskilled |
| Olli | Tonkean | 2 | M | 12 | B | 2/8 | 6/8 | skilful |
| Wallace | Tonkean | 2 | M | 16 | A | 4/8 | 6/8 | unskilled |
| Walt | Tonkean | 2 | M | 15 | B | 3/8 | 4/8 | skilful |
| Yang | Tonkean | 2 | M | 14 | A | 3/8 | 3/8 | unskilled |
| Boo | Capuchin | 3 | M | 3 | A | 2/8 | 3/8 | skilful |
| Conan | Capuchin | 3 | M | 7 | A | 3/8 | 7/8 | skilful |
| Doriana | Capuchin | 3 | F | 6 | A | 4/8 | 4/8 | unskilled |
| Einstein | Capuchin | 3 | M | 6 | B | 2/8 | 5/8 | skilful |
| Falafel | Capuchin | 3 | M | 5 | A | 2/8 | 7/8 | skilful |
| Google | Capuchin | 3 | F | 3 | B | 3/8 | 4/8 | skilful |
| Koli | Capuchin | 3 | F | 10 | B | 4/8 | 0/3^2^ | unskilled |
| Litchi | Capuchin | 3 | F | 14 | B | 2/8 | 5/8 | skilful |
| Willow | Capuchin | 3 | F | 15 | A | 3/8 | 4/8 | unskilled |
| *^1^This subject participated in another social evaluation experiment a few months before (see Hirel et al., 2025)* | | | | | | | | |
| *^2^This subject did only three trials at the Choice test because she left the experimental room in the middle of the session and never came back.* | | | | | | | | |

| 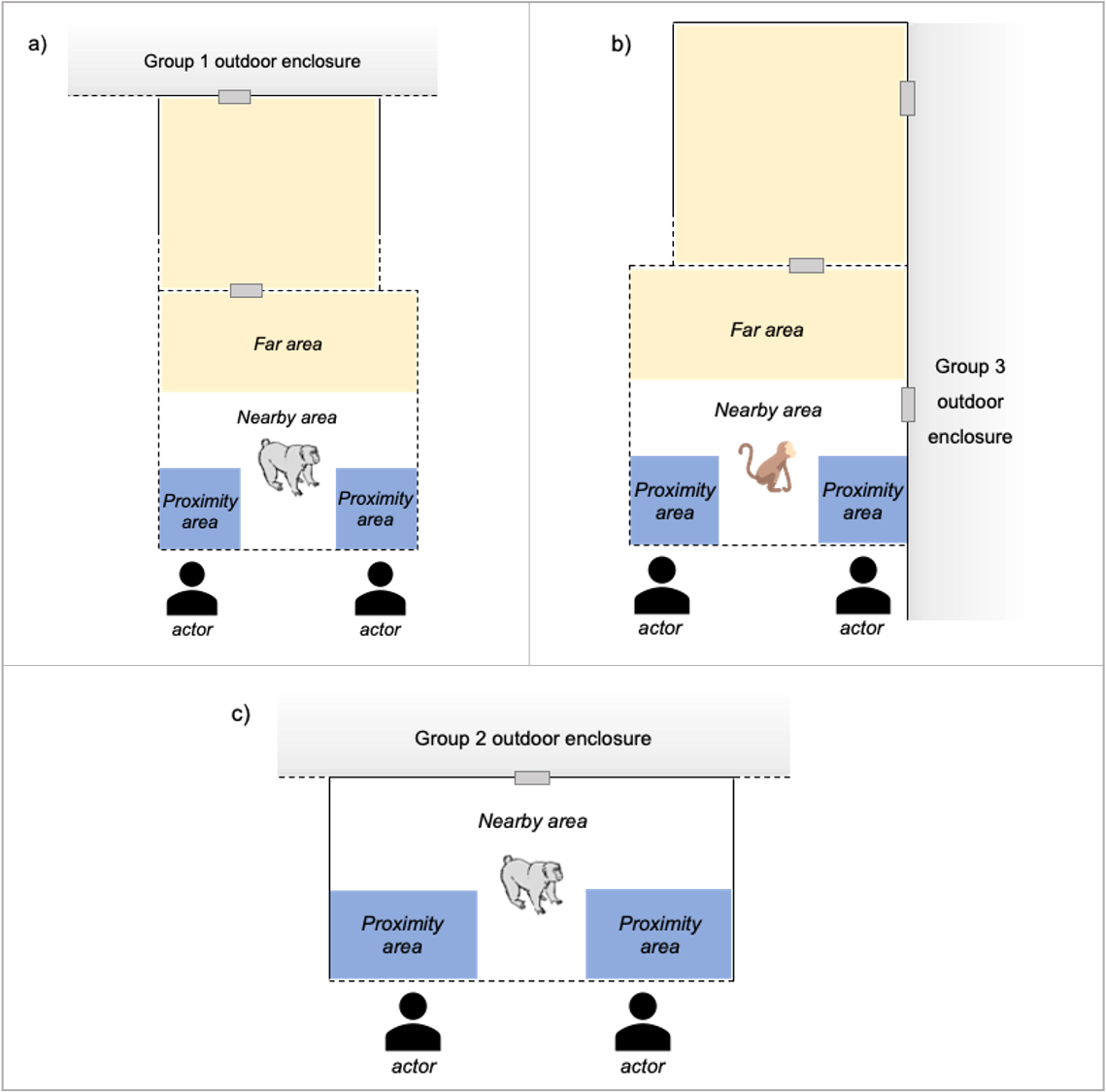 |
| --- |
| Figures S1: Configuration of the experimental rooms where a) the Tonkean macaques of group 1, b) the brown capuchins (group 3), and c) the Tonkean macaques of group 2 were tested. Each experimental room was divided into three areas for coding the subjects’ position (see more information below): two proximity areas in blue, the nearby area in white and the far area in yellow. Note that for group 2, subjects were in the far area only when at the trapdoor or outside but with visual access to the inside of the experimental room, because this experimental room was too small to define a far area in it. |

### Materials

Differently looking containers (in colours of screw caps and in sizes: small: 120 mL, medium: 180 mL, large: 250 mL) were used to emphasise the actors’ (in)ability to open any kind of screw-top containers. Both actors manipulated the same combination of size and coloured screw caps during the demonstration sessions while only the large containers with the yellow screw cap were used for the other experimental steps. Containers were filled with raisins for about ^2^/_3_ of the volume and raisins were used as rewards. At each step, the actors sat at two predetermined locations in front of the mesh of the testing room, 1.5 m apart and with a distance from the mesh adjusted for the arm-length of each subject. The actors carried their containers in identical baskets and, once opened, emptied them into transparent plastic bowls (750 mL). The plastic bowls were placed and removed exclusively by the experimenter, before and after each trial. Both the containers and plastic bowls were out of reach of the subjects. Two identical wooden sticks (length = 30 cm) ending with yellow wooden squares (side = 8 cm) were used as targets.

The same two familiar humans played the roles of the skilful and the unskilled actors for all subjects. Although the actors were a man and a woman, all subjects were highly habituated to both male and female caretakers and experimenters, so this factor was not expected to affect their behaviour in our study. For all experimental steps, each trial started with the two actors moving simultaneously to their designated location (right or left) and placing their basket containing their container(s) next to them (except for the IPA trials which did not involve containers).

### Target check

We verified that all subjects could make optimal choices between two alternatives by touching targets. First, the experimenter ensured that the subject was familiar with touching a target by rewarding the subject with one raisin after each touch on the target presented through the mesh, for four or five times. Then, the experimenter presented the subject with two targets, each above a different food option displayed on small plates on the ground: two raisins on one side (i.e., optimal option) and no food on the other side. The subject could obtain the food option (two raisins or no food) that was below the target they had touched. Sessions of 12 trials were conducted, with the side of the food being pseudo-randomised (i.e., equal number of trials on each side). A subject had to choose the optimal option on at least ten out of 12 trials in a session to be considered successful and be tested for the experiment.

### Familiarisation

The subjects underwent a session to familiarise themselves with the containers and how humans can open them to release the food they contain. To increase the possibility for the subjects to evaluate the actors’ skills at manipulating the containers, we reasoned that the subjects should previously experience the possible states of the containers (i.e., open or closed) and the resulting outcomes in their food intake, and the fact that a skilful human can open and close the containers. The experimenter sat in front of the experimental room in front of the subject and had three closed containers (one of each size) filled with one grape next to her. The experimenter took one container, shook it to draw the subject’s attention, manipulated it for about five seconds, then opened it and showed the opened container, the lid and the grape to the subject, and finally fed the subject with the grape. She put again a grape into the same container, closed it, and repeated once more the same actions. The experimenter then handed the closed baited container to the subject which could freely explore it through the mesh for about 20 seconds or until loss of interest. If the subject managed to open the container, the experimenter let the subject take the food; if not, the experimenter took back the container, and opened it to give the food to the subject. The same procedure was then repeated with the other two containers.

### Demonstration sessions

Each demonstration session began with the two actors moving simultaneously to their designated location, holding a target and placing their basket containing identical sets of baited containers next to them. One transparent bowl was in the middle equidistant from the actors, out of reach for the subject (Figure 1c). A demo trial started with an actor placing four containers aligned in front of them, out of reach for the subject, and then presenting their target. Once the subject had touched the target, the actor started manipulating the four containers. After trying for around five seconds, the skilful actor successfully opened each container, emptied it into the transparent bowl, put the container and the lid back into their basket, and repeated the same actions with the next three containers. The unskilled actor did the same actions, except that they attempted to open the container for around five seconds but failed, and tried in vain to empty the closed container into the transparent bowl.

We are aware that five seconds may appear relatively brief for an unsuccessful attempt; however, it was important to keep the manipulation time equal between successful and unsuccessful attempts, to avoid confounding a potential preference/avoidance for one of the roles with this actor merely being associated with longer handling of the container. As the monkeys’ attention span is relatively short, we opted for the compromise of five seconds to avoid introducing too much additional noise in the data due to a loss of interest by the subjects.

### Video coding

All the videos were coded frame by frame by an observer using Behavioral Observation Research Interactive Software (BORIS v.8.20; Friard & Gamba, 2016). A second observer, who was unaware of the study design and hypothesis, coded independently 38 videos which were pseudo-randomly selected to include 20% of the sessions for each combination of phase, species and subjects, giving a relatively representative sample of each behaviour coded. Inter-coder reliability was calculated using Cohen’s kappa coefficient for actor choices and synchronicity of targets’ presentation, and using Intraclass Correlation Coefficients (ICC; Koo & Li, 2016) for looking time, latency of choices, and the duration of subjects’ position based on a single rating (*k* = 2 raters), two-way mixed-effects model and consistency. We calculated ICCs with the function icc from the package irr (version 0.84.1; Gamer et al., 2019) in R (version 4.3.2; R Core Team, 2022).

For the IPA and Choice Test, videos were coded for: a) the choices of the subjects toward either actor (Cohen’s kappa, K=1, N=80), and b) the synchronicity of targets’ presentation by the actors (Cohen’s kappa: K=0.63, N=80; see Table S2).

For Baseline and Expectation Tests, videos were coded for: a) the duration of looking at the skilful actor (inter-coder reliability: ICC=0.982, N=14) and the unskilled actor (inter-coder reliability: ICC=0.98, N=14), and b) the position of the subjects in the experimental room [close to the skilful actor (inter-coder reliability: ICC=0.993, N=14), close to the unskilled actor (inter-coder reliability: ICC=0.996, N=14), in the ‘nearby’ area (inter-coder reliability: ICC=0.995, N=14), or far away (inter-coder reliability: ICC=1, N=14); see Table S2, Figures S1 & S2].

For the demonstration sessions, videos were coded for: a) the duration of looking at the skilful actor (inter-coder reliability: ICC=0.96, N=26) or the unskilled actor (inter-coder reliability: ICC=0.98, N=26), and b) the position of the subjects in the experimental room [close to the demonstrating actor (inter-coder reliability: ICC=0.94, N=52), in the ‘nearby’ area (inter-coder reliability: ICC=0.893, N=52), or far away ((inter-coder reliability: ICC=0.999, N=52); see Table S2, Figures S1 & S2].

| Table S2: Definitions of coded behaviours | | |
| --- | --- | --- |
| **Behaviour** | **Definition** | **Test phase** |
| Choice | The subject touches one of the targets with hand or mouth. | IPA, Choice Test |
| Target synchronicity | Difference of time between the presentation of target of the actors. The presentation was considered asynchronous when the targets were presented with a difference of more than four frames (i.e., 0.13 seconds). | IPA, Choice Test |
| Looking | Time when the subject’s head (or eyes when visible) was oriented toward an actor (face, body, or hands) or toward the container they held for at least four consecutive frames (i.e., 0.13 seconds). | Demonstration, Baseline, Expectation Tests |
| Position | Time during which the subject (at least the upper half of the body) was in a predefined area of the experimental room (‘proximity’ area, ‘nearby’ area, ‘far’ area; see Figures S1 & S2). | Demonstration, Baseline, Expectation Tests |

| 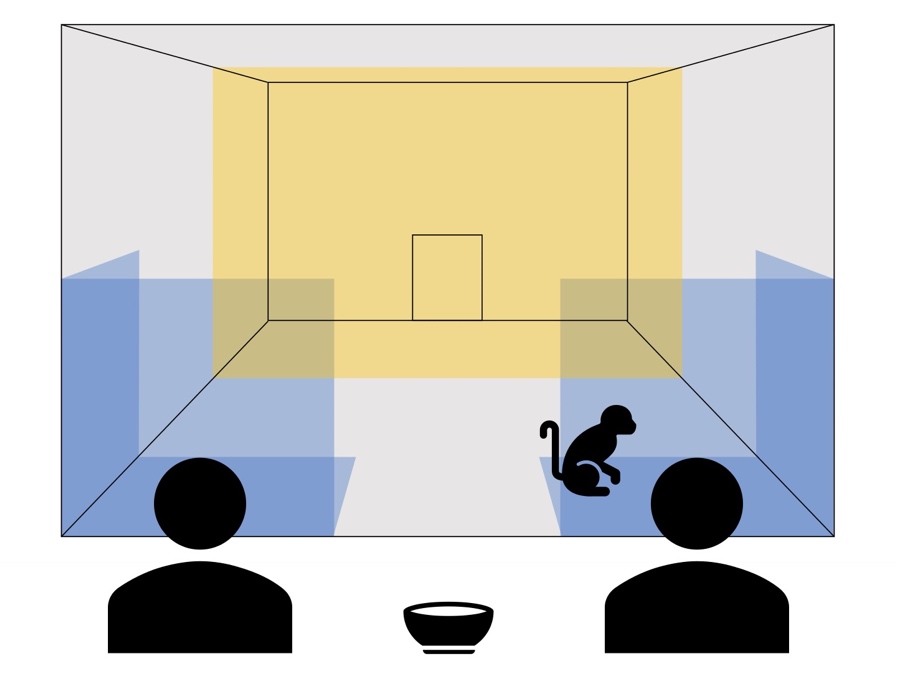 |
| --- |
| Figure S2: Schematic representation of the four areas of the experimental room defined for coding the subject’s position. Four areas in the experimental rooms were delimited horizontally and extended to about 1.5 m vertically: a) two ‘proximity’ areas to the actors which were located in front of the actors and had an area of around 2 m^2^ each (in blue), b) the ‘nearby’ area which was defined as the half of the room closer to the front mesh but not within the proximity areas (in grey), and c) the ‘far’ area which was defined as the half of the room further away from the mesh (in yellow). |

## Data analyses & Results

### Choices in the Choice Test

With this model, we wanted to estimate whether subjects chose the skilful actor more often than the unskilled actor in the Choice Test and whether the probability of optimal choices increased with direct experience (i.e., trial number). We fitted a Generalized Linear Mixed Model (GLMM; Baayen, 2008) with logit link function (McCullagh & Nelder, 1989) and a binomial error structure, using the function glmer of the package lme4 (version 1.1-35.1; Bates et al., 2015) in R (version 4.3.2; R Core Team, 2022). The model included subjects’ choices (1: choice for the skilful actor; 0: choice for the unskilled actor) as the response variable, trial number as the main predictor, species (capuchin, Tonkean), attention directed at the actors during the demonstration (i.e., minimum looking time per subject to the skilful and unskilled partner), and the three-way interaction between these predictors as fixed effects. To control for their potential effects, we also included sex of the subjects, location (left, right) and identity (A, B) of the skilful actor, and synchronicity of targets’ presentation (-1: unskilled actor first, 0: synchronous, +1: skilful actor first). To account for random individual differences, to avoid overconfident model estimates and to keep type I error rate at the nominal level of 5%, we included subject ID as a random intercept effect and all identifiable random slopes within subject, which were trial number, location of the skilful actor, and synchronicity of targets’ presentation (Barr et al., 2013; Schielzeth & Forstmeier, 2009). Correlations between the random intercept and slopes were also included into the model. Prior to fitting the model, trial number was transformed to range from 0 to 1, and demo looking and synchronicity were *z*-transformed to ease interpretation of the model estimates (Schielzeth, 2010) and model convergence. We assessed whether collinearity was an issue by means of Variance Inflation Factors (VIF; Field, 2005), determined for a linear model using the function vif of the package car (version 3.1-2; Fox & Weisberg, 2011). We estimated the stability of the model by dropping the subjects one at a time from the data and comparing the estimates derived for models fitted to these subsets with those obtained for the full data set. We assessed model overdispersion using a function provided by Roger Mundry (Mundry, 2023). The model revealed to be of acceptable stability and with no obvious issue of collinearity (maximum VIF: 2.321).

| Table S3: Average looking time toward the actors while they manipulated the containers during the demonstration sessions for both species. ‘Min looking time’ is the measure of attention used in the model analysis to estimate subjects’ optimal choices. | | | | | |
| --- | --- | --- | --- | --- | --- |
| **Average looking time at each trial** | | | | | |
|  | Target of looking | Mean ± sd | Mean duration of demonstration trial | | Percentage |
| Tonkean macaques | both actors | 20.52 ± 7.33 s | 34.43 s | | 59.8% |
|  | skilful actor | 19.1 ± 6.57 s | 33.71 s | | 56.9% |
|  | unskilled actor | 22 ± 7.82 s | 35.14 s | | 62.8% |
| Brown capuchins | both actors | 13.15 ± 7.84 s | 31.33 s | | 43.5% |
|  | skilful actor | 13.56 ± 7.72 s | 30.07 s | | 45.1% |
|  | unskilled actor | 12.74 ± 8 s | 32.62 s | | 39.1% |
|  |  |  |  | |  |
| **Average looking time in total** | | | | | |
|  | Target of looking | Mean ± sd | | Individual range | |
| Tonkean macaques | both actors | 129.3 ± 30.98 s | | 79.76 s - 197.91 s | |
|  | skilful actor | 122.22 ± 24.54 s | | 79.76 s - 158.69 s | |
|  | unskilled actor | 136.39 ± 36.23 s | | 87.75 s - 197.91 s | |
|  | min looking time | 115.11 ± 25.16 s | | 79.76 s – 158.69 s | |
| Brown capuchins | both actors | 88.43 ± 25.85 s | | 52.14 s - 136.73 s | |
|  | skilful actor | 91.9 ± 26 s | | 57.11 s - 136.73 s | |
|  | unskilled actor | 84.96 ± 26.62 s | | 52.14 s - 130.88 s | |
|  | min looking time | 79.55 ± 24.69 s | | 52.14 s – 120.62 s | |

| 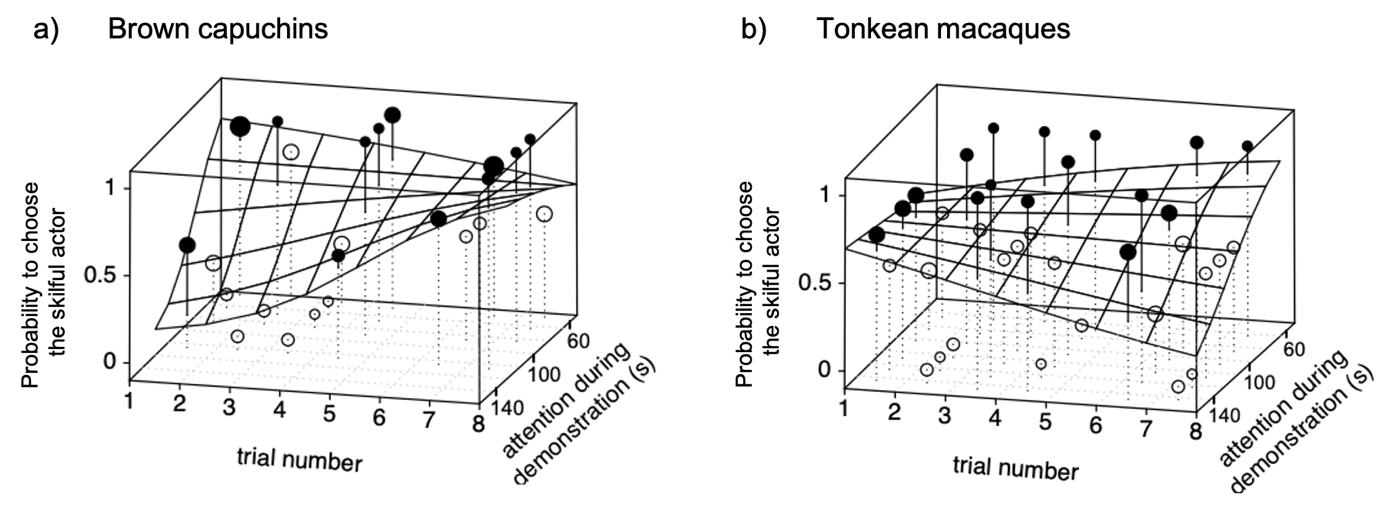 |
| --- |
| Figure S3: Three-dimensional plots showing the results of the logistic model analysing the Choice Test, separately for a) brown capuchins and b) Tonkean macaques. Points depict the average proportion of choices for the skilful actor per cell of the fitted model surface. The size of the points is proportional to the number of observations in the respective cell (range: 1 to 8). Filled and open points lie above and below the fitted model, respectively. In both plots, the fitted values were calculated for all non-plotted predictors being average. The measure of attention during demonstration was the minimum sum of looking time per subject to the skilful and unskilled actor (i.e., the time in seconds during which each subject observed each actor). |

### Shift in preference for the skilful actor

We used a GLMM (Baayen, 2008) with logit link function (McCullagh & Nelder, 1989) and a binomial error structure to estimate the change in preference for the skilful actor during the IPA and the Choice Test. We fitted the model in R (version 4.3.2; R Core Team, 2022) using the function glmer of the package lme4 (version 1.1-35.1; Bates et al., 2015). The response variable was a matrix comprising the number of choices for the skilful and unskilled actor for each subject and phase. In this model, we included phase (IPA, Choice Test) as the main predictor and species (capuchin, Tonkean). To account for random individual differences, while avoiding overconfident model estimates and keeping type I error rate at the nominal level of 5%, we included subject ID as a random intercept effect and the only theoretically identifiable random slope (Barr et al., 2013; Schielzeth & Forstmeier, 2009), which was phase within subject. Correlations between the random intercept and slope were also included in the model.

We estimated the stability of the model by dropping the subjects one at a time from the data and comparing the estimates derived for models fitted to these subsets with those obtained for the full data set using a function provided by Roger Mundry (Mundry, 2023). We assessed whether collinearity among the predictors was an issue by means of Variance Inflation Factors (VIF; Field, 2005), determined for a linear model using the function vif of the package car (version 3.1-2; Fox & Weisberg, 2011). We assessed overdispersion using a function provided by Roger Mundry (Mundry, 2023). The model revealed to be of good stability, was mildly underdispersed (dispersion parameter: 0.690; which may result in too conservative tests), and with no obvious issue of collinearity (maximum VIF: 1). We obtained confidence intervals of model estimates and fitted values by means of a parametric bootstrap (N = 1000 bootstraps; function bootMer of the package lme4). To test the effect of individual fixed effects, we conducted likelihood ratio tests (Dobson & Barnett, 2018) that compared the full models with reduced models, each lacking one fixed effect at a time (Barr et al., 2013).

### WS/LS strategy

We estimated the effect of positive outcomes on the probability to stay with or switch actor choices during the Choice Test. We fitted a GLMM (Baayen, 2008) with logit link function (McCullagh & Nelder, 1989) and a binomial error structure using the function glmer of the package lme4 (version 1.1-35.1; Bates et al., 2015) in R (version 4.3.2; R Core Team, 2022). In the model, we included strategy (stay, switch) as the response variable, reward (yes, no) as the fixed effect predictor of interest, and species as a control predictor. We included subject ID as a random intercept effect and reward as a random slope to account for random individual differences, to avoid overconfident model estimates and to keep type I error rate at the nominal level of 5%. Before fitting the model, we dummy coded and then centred reward to include it in the random effect structure. Correlations between the random intercept and slope were also included in the model.

We assessed whether collinearity among the predictors was an issue by means of Variance Inflation Factors (VIF; Field, 2005), determined for a linear model using the function vif of the package car (version 3.1-2; Fox & Weisberg, 2011). We assessed overdispersion using a function provided by Roger Mundry (Mundry, 2023). We estimated the stability of the model by dropping the subjects one at a time from the data and comparing the estimates derived for models fitted to these subsets with those obtained for the full data set using a function provided by Roger Mundry (Mundry, 2023). The model revealed good stability, was slightly underdispersed (dispersion parameter: 0.87, which may result in too conservative tests), and with no obvious issue of collinearity (maximum VIF: 1). We obtained confidence intervals of model estimates and fitted values by means of a parametric bootstrap (N = 1000 bootstraps; function bootMer of the package lme4).

### Anticipatory behaviours

We estimated the effect of trial (Baseline, ExpT1, ExpT2) on the subjects’ proportion of time looking at and/or in proximity to both actors. We fitted two models with a beta error distribution (McCullagh & Nelder, 1989) in R (version 4.3.2; R Core Team, 2022) using the function glmmTMB of the equally named package (version 1.1.8; Brooks et al., 2017). The two models had exactly the same structure except for the response variable, which was either the proportion of time looking at either actor or the proportion of time spent in proximity to either actor. Actor (skilful, unskilled), trial (Baseline, ExpT1, ExpT2) and their interaction were the main predictors. To control for their potential effects, we also included species, sex of the subjects, and identity of the skilful actor.

To account for random individual differences, to avoid overconfident model estimates and to keep type I error rate at the nominal level of 5%, we included subject ID as a random intercept effect and all theoretically identifiable random slopes (Barr et al., 2013; Schielzeth & Forstmeier, 2009), which were trial and actor within subject. In addition, we included the random intercept effect of trial nested in subject to account for the fact that the data for the skilful and unskilled actor for any given combination of subject and trial were not independent. Originally, we also included parameters of the correlations among random slopes and intercepts, but as the models did not converge, we had to exclude them. Before fitting the models, as the proportion of time looking or spent in proximity ranged from 0 to 0.98 and beta distribution cannot cope with values being exactly zero or one, both response variables were transformed to bring the observations slightly closer to 0.5 (Smithson & Verkuilen, 2006). We also dummy coded and centred phase and actor to include them in the random effect structure.

We estimated the stability of the model by dropping the subjects one at a time from the data and comparing the estimates derived for models fitted to these subsets with those obtained for the full data set using a function provided by Roger Mundry (Mundry, 2023). We assessed whether collinearity among the predictors was an issue using Variance Inflation Factors (VIF; Field, 2005), determined for a linear model using the function vif of the package car (version 3.1-2; Fox & Weisberg, 2011). We assessed overdispersion using a function provided by Roger Mundry (Mundry, 2023). The models were of good stability and with no obvious issue of collinearity (maximum VIF: 1.023). The model for the proportion of time looking was not overdispersed (dispersion parameter: 1.08) but the model for the proportion of time in proximity was mildly overdispersed (dispersion parameter: 1.44, which may result in anti-conservative tests).

As an overall test of the fixed effects and to avoid cryptic multiple testing (Forstmeier & Schielzeth, 2011), we compared these full models with null models lacking the effects of trial, actor and their interaction but being otherwise identical, using likelihood ratio tests (Dobson & Barnett, 2018). To test the effect of individual fixed effects, we conducted likelihood ratio tests (Dobson & Barnett, 2018) that compared the looking full model with reduced models, each lacking one fixed effect at a time (Barr et al., 2013), and we used a function that allows to correct significance tests of individual effects for overdispersion for the proximity model. We obtained confidence intervals of model estimates and fitted values by means of a parametric bootstrap (N = 1000 bootstraps; function simulate of the package glmmTMB).

| Table S4: Average looking time and spatial proximity time towards the actors while they both manipulated one container filled with food simultaneously for 20 seconds during the Baseline and Expectation trials. | | | | | | | | |  |
| --- | --- | --- | --- | --- | --- | --- | --- | --- | --- |
| **Average looking time (s)** | | | | | | | | | |
|  |  | **Tonkean macaques** | | | | **Brown capuchins** | | | **Both species** |
| **Baseline** | skilful actor | 8.87 ± 3.9 | | 44.35% | | 3.91 ± 2.53 | | 19.55% | 6.52 ± 4.11 |
|  | unskilled actor | 6.62 ± 5.82 | | 33.1% | | 4.08 ± 3.14 | | 20.4% | 5.42 ± 4.8 |
| **ExpT1** | skilful actor | 6.8 ± 4.69 | | 34% | | 2.97 ± 3.64 | | 14.85% | 4.99 ± 4.56 |
|  | unskilled actor | 3.5 ± 3.07 | | 17.5% | | 2.3 ± 3.06 | | 11.5% | 2.93 ± 3.04 |
| **ExpT2** | skilful actor | 8.4 ± 3.38 | | 42% | | 3.98 ± 5.46 | | 19.9% | 6.44 ± 4.84 |
|  | unskilled actor | 2.65 ± 2.61 | | 13.25% | | 3.24 ± 4.54 | | 16.2% | 2.91 ± 3.5 |
| **Total** | both actors | 12.3 ± 5.04 | | 20.47% | | 6.81 ± 5.4 | | 11.35% | 9.74 ± 5.85 |
|  |  |  |  | |  | |  | |  |
| **Average spatial proximity time (s)** | | | | | | | | | |
|  |  | **Tonkean macaques** | | | | **Brown capuchins** | | | **Both species** |
| **Baseline** | skilful actor | 3.73 ± 5.67 | | 18.65% | | 2.78 ± 3.84 | | 13.9% | 3.28 ± 4.78 |
|  | unskilled actor | 0.16 ± 0.51 | | 0.8% | | 2.87 ± 4.2 | | 14.35% | 1.44 ± 3.15 |
| **ExpT1** | skilful actor | 3.15 ± 6.41 | | 15.75% | | 2.82 ± 4.5 | | 14.1% | 2.99 ± 5.44 |
|  | unskilled actor | 0.91 ± 2.87 | | 4.55% | | 1.9 ± 3.93 | | 9.5% | 1.38 ± 3.35 |
| **ExpT2** | skilful actor | 4.49 ± 7.56 | | 22.45% | | 4.35 ± 7.86 | | 21.75% | 4.43 ± 7.47 |
|  | unskilled actor | 0 | | 0% | | 2.9 ± 5.76 | | 14.5% | 1.29 ± 3.98 |
| **Total** | both actors | 4.15 ± 6.39 | | 6.92% | | 5.82 ± 6.44 | | 9.7% | 4.92 ± 6.41 |

| Table S5: Results of the full model for the subjects’ proximity time during the Baseline and Expectation trials 1 and 2 (estimates together with standard errors, 95% confidence limits, significance tests and the estimates range obtained when dropping levels of grouping factors one at a time). | | | | | | | | | |
| --- | --- | --- | --- | --- | --- | --- | --- | --- | --- |
| **Term** | **Estimate** | **SE** | **CL_lower_** | **CL_upper_** | **χ^2^** | **df** | ***p*** | **min** | **max** |
| (Intercept) | -1.345 | 0.390 | -2.072 | -0.723 |  |  | ^1^ | -1.58 | -1.191 |
| Phase ExpT1 | -0.113 | 0.430 | -0.821 | 0.575 |  |  | ^1^ | -0.22 | -0.021 |
| Phase ExpT2 | 0.058 | 0.438 | -0.751 | 0.760 |  |  | ^1^ | -0.083 | 0.169 |
| Actor role | -0.283 | 0.429 | -1.066 | 0.424 |  |  | ^1^ | -0.398 | -0.196 |
| Species | -0.240 | 0.259 | -0.705 | 0.241 | 1.231 | 1 | 0.354 | -0.353 | -0.111 |
| Sex | -0.002 | 0.262 | -0.428 | 0.438 | 0 | 1 | 0.993 | -0.143 | 0.129 |
| ID skilful | -0.129 | 0.258 | -0.621 | 0.309 | 0.36 | 1 | 0.618 | -0.269 | -0.044 |
| Phase ExpT1 × actor role | 0.052 | 0.606 | -1.019 | 1.106 | 0.1 | 2 | 0.932 | -0.044 | 0.239 |
| Phase ExpT2 × actor role | -0.108 | 0.614 | -1.088 | 1.035 |  |  | ^2^ | -0.261 | 0.058 |
| *Phase, actor role, species, sex and ID of the skilful actor were dummy coded with their reference level being respectively baseline, skilful, capuchins, female and actor A.*  *^1^not indicated because of very limited interpretability.*  *^2^only one* p*-value because the interaction was tested as a whole.* | | | | | | | | | |
|  |  |  |  |  |  |  |  |  |  |

## References

Baayen, R. H. (2008). *Analyzing Linguistic Data*. Cambridge University Press. https://doi.org/10.1017/CBO9780511801686

Barr, D. J., Levy, R., Scheepers, C., & Tily, H. J. (2013). Random effects structure for confirmatory hypothesis testing: Keep it maximal. *Journal of Memory and Language*, *68*(3), 255–278. https://doi.org/10.1016/j.jml.2012.11.001

Bates, D., Mächler, M., Bolker, B., & Walker, S. (2015). Fitting linear mixed-effects models using lme4. *Journal of Statistical Software*, *67*(1), 1–48. https://doi.org/10.48550/arXiv.1406.5823

Brooks, M. E., Kristensen, K., van Benthem, K. J., Magnusson, A., Berg, C. W., Nielsen, A., Skaug, H. J., Machler, M., & Bolker, B. M. (2017). glmmTMB balances speed and flexibility among packages for zero-inflated generalized linear mixed modeling. *The R Journal*, *9*(2), 378–400. https://doi.org/10.3929/ethz-b-000240890

Dobson, A. J., & Barnett, A. G. (2018). *An introduction to generalized linear models: Vol. 4th ed.* Chapman & Hall/CRC. https://doi.org/10.1201/9781315182780

Field, A. (2005). *Discovering Statistics using SPSS*. Sage.

Forstmeier, W., & Schielzeth, H. (2011). Cryptic multiple hypotheses testing in linear models: Overestimated effect sizes and the winner’s curse. *Behavioral Ecology and Sociobiology*, *65*(1), 47–55. https://doi.org/10.1007/s00265-010-1038-5

Fox, J., & Weisberg, S. (2011). *An R companion to applied regression: Vol. second edition*. Sage.

Friard, O., & Gamba, M. (2016). BORIS: A free, versatile open-source event-logging software for video/audio coding and live observations. *Methods in Ecology and Evolution*, *7*(11), 1325–1330. https://doi.org/10.1111/2041-210X.12584

Gamer, M., Lemon, J., Fellows, I., & Singh, P. (2019). *irr: Various Coefficients of Interrater Reliability and Agreement*. https://doi.org/10.32614/CRAN.package.irr

Hirel, M., Meunier, H., Mundry, R., Rakoczy, H., Fischer, J., & Keupp, S. (2025). Choose Your Partner: Social Evaluation of Skillfulness at Cooperative Co-Action Tasks in Tonkean Macaques (Macaca tonkeana). *Animal Behavior and Cognition*, *12*(3), 330–359. https://doi.org/10.26451/abc.12.03.02.2025

Koo, T. K., & Li, M. Y. (2016). A Guideline of Selecting and Reporting Intraclass Correlation Coefficients for Reliability Research. *Journal of Chiropractic Medicine*, *15*(2), 155–163. https://doi.org/10.1016/j.jcm.2016.02.012

McCullagh, P., & Nelder, J. A. (1989). *Generalized Linear Models* (2nd ed.). Chapman & Hall. https://doi.org/10.1201/9780203753736

Mundry, R. (2023). *Some R functions* [Computer software]. Zenodo. https://doi.org/10.5281/zenodo.7670524

R Core Team. (2022). R: A language and environment for statistical computing. *R Foundation for Statistical Computing, Vienna, Austria*. https://www.r-project.org/

Schielzeth, H. (2010). Simple means to improve the interpretability of regression coefficients. *Methods in Ecology and Evolution*, *1*(2), 103–113. https://doi.org/10.1111/j.2041-210X.2010.00012.x

Schielzeth, H., & Forstmeier, W. (2009). Conclusions beyond support: Overconfident estimates in mixed models. *Behavioral Ecology*, *20*(2), 416–420. https://doi.org/10.1093/beheco/arn145

Smithson, M., & Verkuilen, J. (2006). A better lemon squeezer? Maximum-likelihood regression with beta-distributed dependent variables. *Psychological Methods*, *11*(1), 54–71. https://doi.org/10.1037/1082-989X.11.1.54
